# Natural variations of HSFA2 enhance thermotolerance in grapevine

**DOI:** 10.1093/hr/uhac250

**Published:** 2022-11-10

**Authors:** Xinna Liu, Haiyang Chen, Shenchang Li, David Lecourieux, Wei Duan, Peige Fan, Zhenchang Liang, Lijun Wang

**Affiliations:** Beijing Key Laboratory of Grape Science and Enology and Key Laboratory of Plant Resources, Institute of Botany, Chinese Academy of Sciences, Beijing 100093, China; China National Botanical Garden, Beijing 100093, China; University of Chinese Academy of Sciences, Beijing 100049, China; Beijing Key Laboratory of Grape Science and Enology and Key Laboratory of Plant Resources, Institute of Botany, Chinese Academy of Sciences, Beijing 100093, China; China National Botanical Garden, Beijing 100093, China; University of Chinese Academy of Sciences, Beijing 100049, China; Beijing Key Laboratory of Grape Science and Enology and Key Laboratory of Plant Resources, Institute of Botany, Chinese Academy of Sciences, Beijing 100093, China; China National Botanical Garden, Beijing 100093, China; University of Chinese Academy of Sciences, Beijing 100049, China; EGFV, Bordeaux Sciences Agro, INRAE, ISVV, Bordeaux University, Villenave d'Ornon F-33882, France; Beijing Key Laboratory of Grape Science and Enology and Key Laboratory of Plant Resources, Institute of Botany, Chinese Academy of Sciences, Beijing 100093, China; China National Botanical Garden, Beijing 100093, China; Beijing Key Laboratory of Grape Science and Enology and Key Laboratory of Plant Resources, Institute of Botany, Chinese Academy of Sciences, Beijing 100093, China; China National Botanical Garden, Beijing 100093, China; Beijing Key Laboratory of Grape Science and Enology and Key Laboratory of Plant Resources, Institute of Botany, Chinese Academy of Sciences, Beijing 100093, China; China National Botanical Garden, Beijing 100093, China; Beijing Key Laboratory of Grape Science and Enology and Key Laboratory of Plant Resources, Institute of Botany, Chinese Academy of Sciences, Beijing 100093, China; China National Botanical Garden, Beijing 100093, China

## Abstract

Heat stress limits growth and development of crops including grapevine which is a popular fruit in the world. Genetic variability in crops thermotolerance is not well understood. We identified and characterized heat stress transcription factor *HSFA2* in heat sensitive *Vitis vinifera* ‘Jingxiu’ (named as *VvHSFA2*) and heat tolerant *Vitis davidii* ‘Tangwei’ (named as *VdHSFA2*). The transcriptional activation activities of VdHSFA2 are higher than VvHSFA2, the variation of single amino acid (Thr315Ile) in AHA1 motif leads to the difference of transcription activities between VdHSFA2 and VvHSFA2. Based on 41 *Vitis* germplasms, we found that *HSFA2* is differentiated at coding region among heat sensitive *V. vinifera*, and heat tolerant *Vitis davidii* and *Vitis quinquangularis*. Genetic evidence demonstrates VdHSFA2 and VvHSFA2 are positive regulators in grape thermotolerance, and the former can confer higher thermotolerance than the latter. Moreover, VdHSFA2 can regulate more target genes than VvHSFA2. As a target gene of both VdHSFA2 and VvHSFA2, overexpression of MBF1c enhanced the grape thermotolerance whereas dysfunction of MBF1c resulted in thermosensitive phenotype. Together, our results revealed that *VdHSFA2* confers higher thermotolerance than *VvHSFA2*, and MBF1c acts as their target gene to induce thermotolerance. The *VdHSFA2* may be adopted for molecular breeding in grape thermotolerance.

## Introduction

Heat stress (HS), as one of the main abiotic stresses, limits the yield of crops. With climate change, extreme high temperature becomes one of the major adverse environmental conditions that plants often encounter [1]. Heat stress generally impairs photosynthetic activity, reduces water content, and has negative effects on cell division and growth [2]. Heat stress can affect many physiological processes of plants, such as growth, development, and reproduction [3]. As sessile organisms, plants have to reprogram their transcripts, proteins, and metabolites to some extent in order to survive in heat stress conditions [3–6].

Heat stress response (HSR) is highly complicated in plants. Current knowledge of plant response to HS mainly includes heat stress transcription factors (HSFs), protein homeostasis, and reactive oxygen species (ROS) [1]. HSFs are the core regulatory factors for HSR and thermotolerance, and HSFs are required for the induction of major HS-induced genes such as *HSPs* [4, 7–9]. HSFs are not only involved in heat stress but also other abiotic stresses including drought, cold, salt, as well as plant growth and development [10]. The number of HSF members varied among plants, with 19 in grape (*Vitis vinifera*), 21 in Arabidopsis (*Arabidopsis thaliana*), and up to 56 in wheat (*Triticum aestivum*) [4, 10, 11]. The basic structure and promoter recognition of HSFs were highly conserved in eukaryotes although HSFs were different in size and sequence [4, 12]. A typical HSF structure generally contains DNA binding domain (DBD), oligomerization domain (OD) or HR-A/B, nuclear localization signal domain (NLS), C-terminal activation domain (CTAD), and nuclear export signal domain (NES) [4, 12, 13]. The DBD contains three α-helix bundle and an antiparallel four-stranded β-sheet, CTAD comprises some aromatic/hydrophobic/acidic (AHA) motifs [13, 14]. Different HSFs members had their specific roles in HSR despite the high similarity among HSF orthologs [15]. HSFA2 acts as key regulator involved in cellular response to various types of environmental stress. Heat stress can induce the expression of HSFA2, and HSFA2 promoted the expression of heat shock protein (HSP) genes and improved acquired thermotolerance of Arabidopsis [16]. The transcription level of HSFA2 was the highest among all 21 Arabidopsis HSFs after heat stress, and overexpression of the Arabidopsis HSFA2 gene improved plant abiotic stress such as heat resistance and salt/osmotic stress tolerance [17]. In tomato, loss function of HSFA2 reduced the viability and germination rate of pollen after heat stress during the stages of meiosis and microspore formation [15]. Overexpression of *ZmHSFA2* in Arabidopsis improved the expression of Arabidopsis raffinose synthase gene, accompanied with increased raffinose content and plant thermotolerance [18]. These findings suggested an extensive role for HSFs in heat tolerance. In grapevine, VvHSFA2 may activate GOLS1 to induce thermotolerance, but there was no genetic evidence of HSFA2 in thermotolerance [19]. What is more, there are hardly any reports that allelic variations of HSFs result in thermotolerance difference in different plant species (or accessions), especially for HSFA2. In addition, exactly how HSFA2 regulates the downstream genes and contributes to heat tolerance in plants including grapevines remains to be explored.

MBF1, multiple bridge factor 1, is a highly conserved transcriptional coactivator that played a crucial role in development process and stress responses [20]. MBF1 contained two parts, N-terminal domain (named MBF1 domain) and C-terminal helix–turn–helix domain [20]. MBF1 was firstly isolated in silkworm, as a bridge between FTZ-F1 and TBP, mediating transactivation by FTZ-F1 [21]. Subsequent studies about MBF1 were performed in yeast [22]. Three MBF1 isoforms exist in the genome of Arabidopsis. MBF1a and MBF1b are involved in the regulation of various developmental processes, while MBF1c is a player of responses to different stress such as salinity and heat [23–27]. In tomato, the transcription level of MBF1-like protein ER24 is up-regulated upon ethylene treatment [28]. Recently, MBF1c were more reported in response to heat stress, such as in wheat, Chinese kale and lily [29–31]. In grape, there is some research about *MBF1a*, which is related to drought stress and high temperature [32, 33]. Rienth *et al.* reported that *VvMBF1c* showed an induction in grape response to high temperature [33, 34]. However, the function of MBF1c is not validated in grapevine, and which transcriptional factor directly modulates MBF1c remains unknown in plants.

Grape (*V. vinifera* L.) is a very popular fruit in the world and with important economic value. However, heat stress can have harmful effects on grape quality and production. With the publishing of the grape genome, genes related to many quality traits such as anthocyanin, resveratrol, sugar, cold, and dehydration have been identified [35–39]. These studies provided information for the study of grape quality formation. Information on grape heat stress is limited although there are some studies about morphological and physiological changes in response to heat stress. Nevertheless, a growing body of omics data provided novel insights for grape heat stress response [5, 40]. Our previous study pointed to the fact that the expression of HSFA2 was increased at transcription and protein level after heat stress, indicating that HSFA2 may play a potential role in grape thermotolerance [5]. However, we lacked genetic evidence of HSFA2 inducing thermotolerance in grapevines. *V. vinifera* accessions that people often consume are sensitive to high temperature, while *Vitis davidii* and *Vitis quinquangularis* appear more thermotolerant than *V. vinifera* [41, 42]. Therefore, it is also very much worth exploring the mechanism of HSFA2 among these grape accessions displaying different thermotolerant capacity.

In this study, we reported the identification and characterization of *HSFA2* gene, which plays a key role in thermotolerance in grapevine based on genetic evidence. *HSFA2* is differentiated at the coding region among heat sensitive *V. vinifera*, and heat tolerant *Vitis davidii* and *Vitis quinquangularis*. The difference in sequences between *VvHSFA2* and *VdHSFA2* may contribute to the different thermotolerance among grape accessions. Moreover, VdHSFA2 can regulate more target genes than VvHSFA2. *MBF1c*, as the target gene of HSFA2, plays a large role in thermotolerance in grapevines. Our results indicate that natural variations of *HSFA2* enhance thermotolerance in *Vitis*.

## Results

### Expression of *HSFA2* in *V. vinifera* and *V. davidii* grapevine leaves in response to high temperatures

**Figure 1:**
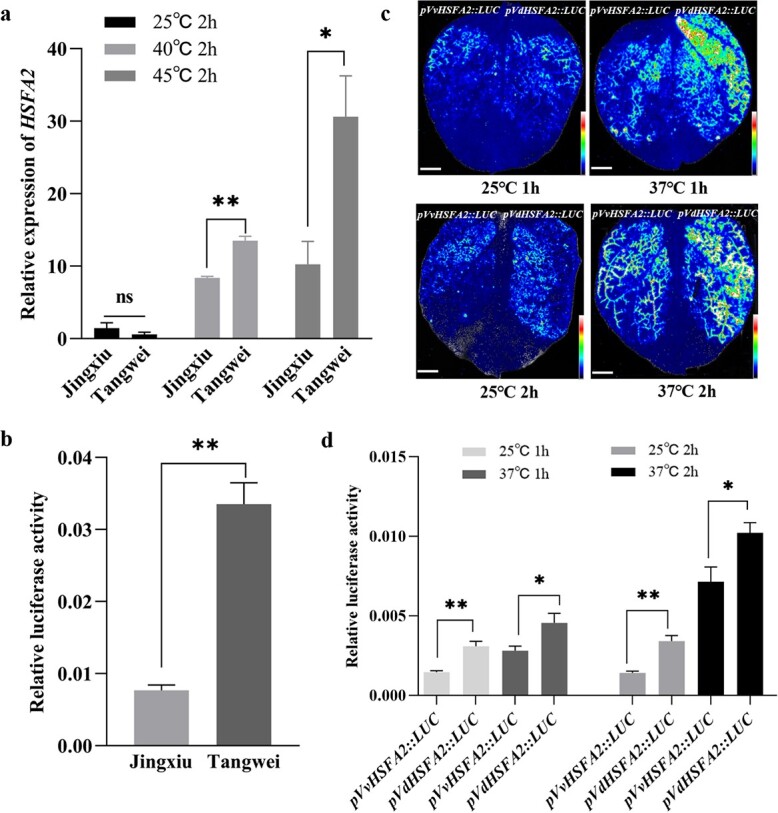
The expression and promoter activities of *Vitis vinifera* ‘Jingxiu’ and *Vitis davidii* ‘Tangwei’ grapevines *HSFA2* under different temperatures. **a** Expression analysis of *HSFA2* in grapevine leaves was conducted using RT-PCR. **b***HSFA2* promoter activities were examined by transient expression assay in *Arabidopsis* protoplasts. Relative Luc/Ren value was measured. **c***HSFA2* promoter activities were examined by transient expression assay in *Nicotiana benthamiana* under room temperature and heat stress. Agrobacterium containing the *VvHSFA2* and *VdHSFA2* promoter vectors were transformed into *N. benthamiana* leaves, respectively. Some transformed lines were treated at 37°C for 1 h or 2 h, and some allowed to still in growth chamber (25°C). A Tanon 5200 Multi luminometer (China) was used to observe fluorescence signal. Bar: 5 mm. **d** Relative Luc/Ren values were measured by transient expression assay in *N. benthamiana* under room temperature (25°C) and heat stress (37°C). *pVvHSFA2* indicates the *HSFA2* promoter from *V. vinifera* ‘Jingxiu’; *pVdHSFA2* indicates the *HSFA2* promoter from *Vitis davidii* ‘Tangwei’. Data are based on three independent biological replicates. Student’s *t*-test was used to determine significant differences (^*^P < 0.05; ^**^P < 0.01).

Our previous study indicated that HSFA2 may play an important role in grape response to heat [5, 19]. Based on these results, we further explored the expression of *HSFA2* in heat sensitive ‘Jingxiu’ (*V. vinifera*) and heat tolerant ‘Tangwei’ (*V. davidii*) grapevines in response to heat stress. After 40°C and 45°C treatments for 2 h, the expression level *HSFA2* in leaves significantly (*P* < 0.05, the same as below) increased when compared to control condition (25°C). Additionally, *HSFA2* showed higher expression in ‘Tangwei’ than in ‘Jingxiu’ upon heat stress (Fig. 1a).

### Sequences and activities comparison of *VvHSFA2* and *VdHSFA2* promoters

The *HSFA2* isoforms from ‘Jingxiu’ and ‘Tangwei’ were named as *VvHSFA2* and *VdHSFA2*, respectively. Two kb of their corresponding promoter regions were cloned and sequenced. Alignment of *VvHSFA2* and *VdHSFA2* promoters revealed 44 single nucleotide polymorphisms (SNPs) and 5 Indels (Fig. S1, see online supplementary material). The Plant CARE database (http://bioinformatics.psb. ugent.be/webtools/plantcare/html/) was used to search conserved motifs. The Plant CARE search revealed that *VvHSFA2* and *VdHSFA2* promoters include 32 common motifs whereas five motifs were present only in the *VvHSFA2* promoter (Fig. S2, see online supplementary material). We conducted transient expression assay in Arabidopsis protoplasts to evaluate *VvHSFA2* and *VdHSFA2* promoter activities. As shown in Fig. 1b, the relative activity of the *VdHSFA2* promoter was higher than the one from the *VvHSFA2* promoter. The promoter activity of *VvHSFA2* and *VdHSFA2* was further compared in the tobacco leaf assay under room temperature (25°C) and heat stress (37°C). As shown in Fig. 1c and d, the HSFA2 promoter activity increased after 37°C heat treatments. In addition, the activity of *VdHSFA2* promoter was higher than *VvHSFA2* promoter under room temperature (25°C) or heat stress (37°C).

**Figure 2:**
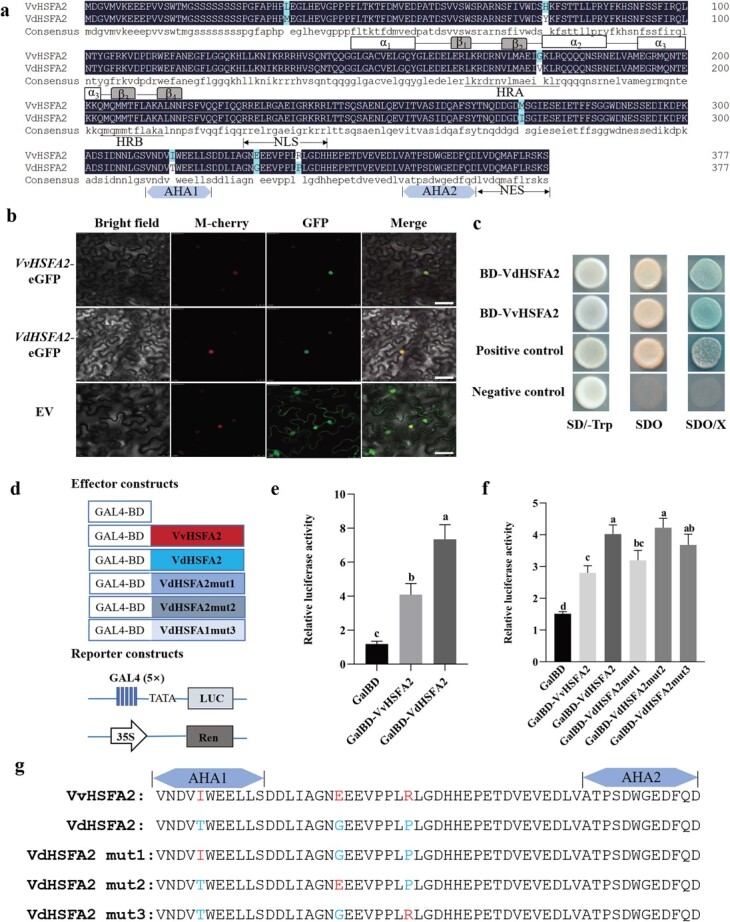
Conserved motif, subcellular localization, and transcriptional activities of HSFA2 from *Vitis vinifera* ‘Jingxiu’ (VvHSFA2) and *Vitis davidii* ‘Tangwei’ (VdHSFA2). **a** Conserved domain analysis of VvHSFA2 and VdHSFA2 amino acid sequences. Conserved domain is shown in the bottom of the sequence. DNA binding domain (DBD) contains three α-helix bundle and a small four-stranded antiparallel β-sheet; oligomerization domain (OD) contains HRA and HRB; NLS, nuclear localization signal; NES, nuclear export signal; C-terminal activation domains (CTAD) are rich in aromatic, hydrophobic, and acidic amino acid residues, so CTAD is also called AHA. **b***VvHSFA2*-eGFP and *VdHSFA2*-eGFP were transiently transformed into epidermal cells of *N. benthamiana* leaves, empty vector (EV) as control. Images under bright field (left), fluorescence (middle), and the merged images are shown on the right. Bar: 20 μm. **c** Analysis of VvHSFA2 and VdHSFA2 transcriptional activity in yeast. The coding sequences of VvHSFA2 and VdHSFA2 were fused with pGBDK7 vector including Gal4 binding domain (BD), respectively. Constructed vector was transformed into yeast strain Y2HGold. The transformed yeast was grown on SD/−Trp, SD/−Trp/-His/−Ade (SDO) and SD/−Trp/-His/−Ade/X-α-gal (SDO/X). Combinations of AD-T with BD-p53 and BD-lam were used as positive and negative control, respectively. **d** Schematic diagrams indicate constructed plasmids in the transient expression assays in Arabidopsis protoplasts. The CDS of *VvHSFA2*, *VdHSFA2*, and *VdHSFA2* mutation were cloned into CTB7-GAL4BD vector as the effector, respectively. Negative control was the empty vector. LUC was used as the reporter to detect the transcriptional activation of VvHSFA2, VdHSFA2, and *VdHSFA2* mutation. Ren was as internal reference. **e** Transcriptional activity analysis of VvHSFA2 and VdHSFA2 in *Arabidopsis* protoplasts by dual luciferase assay. Constructed vector was co-transformed *Arabidopsis* protoplasts with firefly LUC reporter vector and the renilla LUC vector. GalBD was used as negative control. **f** Comparison of transcriptional activity of VvHSFA2, VdHSFA2, and VdHSFA2 mutation in *Arabidopsis* protoplasts by dual luciferase assay. **g** Partial diagrams of VvHSFA2, VdHSFA2, and VdHSFA2 mutation are used in **f**. Amino acid mutant was shown in red characters in VdHSFA2 mutation. VdHSFA2-mutant1 indicates that the 315 th amino acid ‘T(Thr)’ (distributing in the region of AHA1) was changed into ‘I (Ile)’ in VdHSFA2 protein through base mutant; VdHSFA2-mutant2 indicates that the 329 th amino acid ‘G (Gly)’ was changed into ‘E(Glu)’ in the VdHSFA2 protein; VdHSFA2-mutant3 indicates that the 336 th amino acid ‘P(Pro)’ was changed into ‘R(Arg)’ in the VdHSFA2 protein. Data are based on three independent biological replicates. Duncan’s test was used to determine significant differences (*P* < 0.05).

### Sequence analysis, subcellular localization, and transcriptional activity of VvHSFA2 and VdHSFA2

The CDS of *HSFA2* were obtained from leaves of ‘Jingxiu’ and ‘Tangwei’, respectively. Sequence analysis revealed that both *VvHSFA2* and *VdHSFA2* transcripts are 1134 bp, which encode 377 amino acids containing a putative HSF domain. *VvHSFA2* and *VdHSFA2* have 11 SNPs (Fig. S3, see online supplementary material) leading to differences for seven amino acid residues distributed in DBD, HR-A/B, and AHA domains (Fig. 2a). Phylogenetic analysis of the VvHSFA2 and VdHSFA2 proteins and their orthologs in various plants was conducted. The phylogenetic analysis was constructed based on the amino acid sequence alignments from representative species. The results showed that VvHSFA2 and VdHSFA2 share the highest sequence identity, followed by MdHSFA2 (*Malus domestica* HSFA2) (Fig. S4, see online supplementary material).

Next, we explored the subcellular localization of VvHSFA2 and VdHSFA2. *VvHSFA2*-GFP and *VdHSFA2*-GFP fusion constructs were transfected into *N. benthamiana* leaves, respectively. Fluorescence analysis of *N. benthamiana* overexpressing *VvHSFA2*-GFP and *VdHSFA2*-GFP indicated nuclear localization, while the GFP control fluorescence was evenly distributed throughout the cell (Fig. 2b).

To explore whether VvHSFA2 and VdHSFA2 possess transcriptional activity, *VvHSFA2* and *VdHSFA2* were constructed into the pGBKT7 vector with the Gal4 DNA binding domain (BD), respectively. The BD-VvHSFA2, BD-VdHSFA2 plasmids and empty vector BD were expressed individually into yeast strain Y2HGold. As shown in Fig. 2c, yeast cells carrying BD-VvHSFA2, BD-VdHSFA2 and the positive control exhibited alpha-galactosidase activity, indicating that VvHSFA2 and VdHSFA2 possess transcriptional activity in yeast. A similar assay was conducted using a dual-luciferase reporter assay in *Arabidopsis* protoplasts. The effector and reporter constructs are shown in Fig. 2d. As shown in Fig. 2e, both VvHSFA2 and VdHSFA2 triggered luciferase activity, in higher proportion for VdHSFA2 when compared to VvHSFA2. To further explore which SNP may explain the different transactivation properties observed between VvHSFA2 and VdHSFA2, we muted three different SNPs located in AHA1 and AHA1-adjacent domains of VdHSFA2 into VvHSFA2 in turn. The muted VdHSFA2 were named as VdHSFA2mut1, VdHSFA2mut2, VdHSFA2mut3 in order of the location of the three SNPs (Fig. 2g). The three muted VdHSFA2 with Gal4 DNA binding domain were co-transformed into *Arabidopsis* protoplasts with LUC and TRL plasmid, respectively. The results showed that only VdHSFA2mut1 (Thr^315^ → Ile^315^), resulting from the nucleotide polymorphism of C/T at the 944th base of HSFA2 coding region, caused significant lower transcriptional activity when compared to VdHSFA2 wild type (Fig. 2f).

To explore whether heat tolerance is correlated with the VdHSFA2mut1 in grape, we measured the heat tolerance of 41 grape accessions (Table S1, see online supplementary material), and combined cloning and sequencing of *HSFA2* isoforms from each germplasm. As shown in Table 1, allele (Ile^315^) was possessed in 38 *V. vinifera* accessions which are heat sensitive (lower F_v_/F_m_ values). However, allele (Thr^315^) was possessed in two *V. davidii* accessions and one *V. quinquangularis* accession which are heat tolerant (higher F_v_/F_m_ values).

**Table 1:** Association of SNPs in *HSFA2* with heat tolerance in grapevines.

Species	SNP1 +100	SNP2 +229	SNP3 +527	SNP4 +819	SNP5 +944	SNP6 +986	SNP7 +1007	No. of accessions	Average F_v_/F_m_
*Vitis vinifera*	T	C	G	G	T	A	G	38	0.26
*Vitis davidii, Vitis quinquangularis*	A	T	T	C	C	G	C	3	0.60^**^

Note: SNP1–7 indicates the variation of *HSFA2* CDS, and their positions relative to ATG are shown. SNP5 located in AHA1 is highlighted. Accessions list of *V. vinifera*, *V. davidii* and *V. quinquangularis* are shown in Table S1 (see online supplementary material). Student’s *t*-test was used to determine significant differences (**P < 0.01). F_v_/F_m_ indicates the thermotolerance of *Vitis*.

### Effect of *VvHSFA2* and *VdHSFA2* on heat tolerance in grape

**Figure 3:**
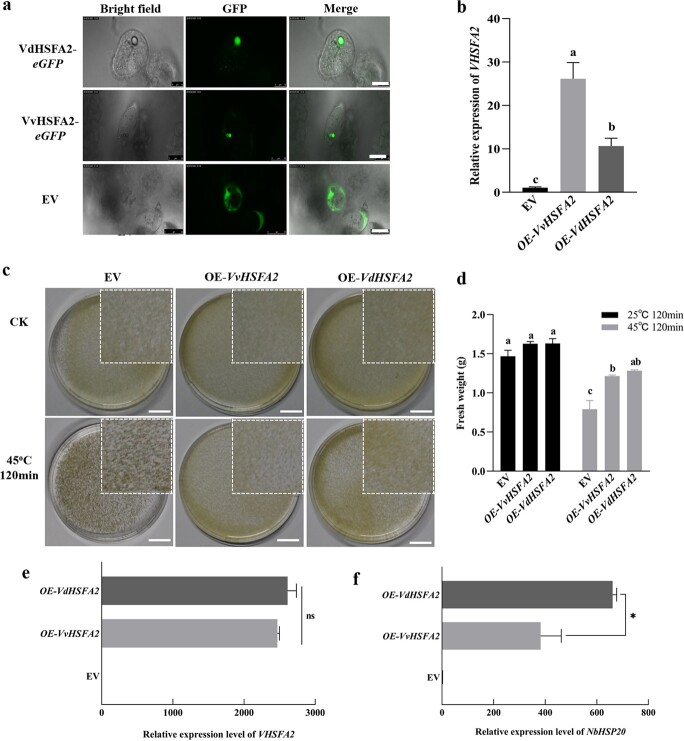
Overexpression of *VvHSFA2* and *VdHSFA2* improved heat tolerance in grape. **a** Detection of transgenic grape suspension cells by GFP fluorescence. The cells transformed with empty vector (EV) was as control. Bar: 20 μm. **b** Expression analysis of *HSFA2* in transgenic grape suspension cells of empty vector (EV), overexpressing *VvHSFA2* (OE-*VvHSFA2*), and overexpressing *VdHSFA2* (OE-*VdHSFA2*) using RT-PCR. **c** Phenotypes of grape suspension cells of EV, OE-*VvHSFA2*, and OE-*VdHSFA2* after 45°C for 2 h and 25°C for 7 d. The grape suspension cells grown under 25°C were as control. The cells in white box lines indicate a zoom on the center sector of the petri dishes. Bar: 1.5 cm. **d** Fresh weight of transgenic grape suspension cells of EV, OE-*VvHSFA2*, and OE-*VdHSFA2* was measured after 45°C for 2 h and 25°C for 7 d. **e** Transgenic lines of similar expression levels of *VvHSFA2* and *VdHSFA2* were acquired in *N. benthamiana*. **f***NbHSP22.0* expression levels in corresponding transgenic lines in **e**. Data are based on three independent biological replicates. Duncan test (*P* < 0.05) or Student’s *t*-test (^*^P < 0.05) were used to determine significant differences. ns: not significant.

To investigate the function of *HSFA2*, transgenic grape suspension cells overexpressing either *VvHSFA2* (OE-*VvHSFA2*) or *VdHSFA2* (OE-*VdHSFA2*) were produced using 35S promoter-driven HSFA2 constructs. Successfully transformed cells were identified by eGFP fluorescence and RT-qPCR (Fig. 3a and b).

Compared with controls (grape cells transformed with empty vector (EV)), *HSFA2* expression was up-regulated ~26 fold and ~ 10 fold in *VvHSFA2* and *VdHSFA2* overexpressing lines, respectively (Fig. 3b). The time for heat stress was the 6th day after subculturing. Then, we evaluated the thermotolerance of control and transgenic grape suspensions by determining their phenotype and fresh weight [43]. The appearance of OE-*VvHSFA2* and OE-*VdHSFA2* grape suspension cells was similar with EV cells when they grew in control conditions at 25°C (Fig. 3c). However, control cells (EV) became black after incubation to 45°C for 2 h and recovery at 25°C for 7 d (Fig. 3c). As shown in Fig. 3d, the fresh weight of EV and transgenic suspension cells all declined significantly after heat stress and recovery, but in lower proportion for HSFA2 transgenic cells than EV cells. Therefore, the overexpression of VvHSFA2 and VdHSFA2 significantly improved heat tolerance of grape, with a better effect of VdHSFA2. In addition, we transiently overexpressed *VvHSFA2* and *VdHSFA2* in grape tissue culture plantlets. Similarly, the results showed that the overexpression of VvHSFA2 and VdHSFA2 significantly improved heat tolerance of grape plants (Fig. S6, see online supplementary material).

To further compare the regulation ability between *VvHSFA2* and *VdHSFA2*, we overexpressed *VvHSFA2* and *VdHSFA2* in *N. benthamiana* transiently. We chose three transgenic *N. benthamiana* lines of OE-*VvHSFA2* and OE-*VdHSFA2,* respectively, showing similar *HSFA2* expression level (Fig. 3e). *HSP22.0* gene was shown to be transactivated by *HSFA2* [17, 44], accordingly, we measured the *HSP22.0* expression level. The results showed that *HSP22.0* expression levels were enhanced in both *N. benthamiana* OE-*VdHSFA2* and OE-*VvHSFA2* lines; however, the last one appeared to a lesser extent (Fig. 3f).

**Figure 4:**
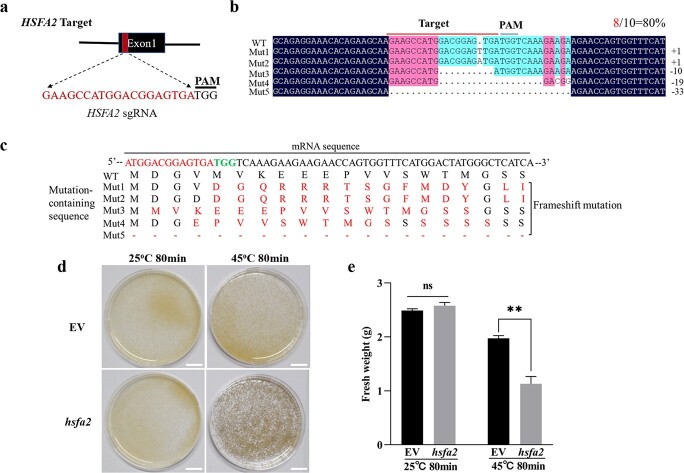
Knockout of *HSFA2* decreased heat tolerance in grape. **a** Diagram illustration of *HSFA2* target sites designed using CRISPR/Cas9 technology. The sequences of sgRNA are shown in red characters, PAM are signed in black. **b** Identification of *HSFA2* knockout mutant (*hsfa2*). Ten colonies for target were analysed. The number of mutant sequences is shown in red. Sequencing results on both sides of the target site were shown. The wild type (WT) and mutant (mut) are shown on the left. Mutant types are shown on the right. **c** Mutations of amino acids in corresponding mutant sequencing in **b**. **d** Phenotypes of grape suspension cells of EV and *hsfa2* mutant after heat treatments (45°C for 80 min) and 25°C for 7 d. The grape suspension cells grown under 25°C were as control. Bar: 1.5 cm. **e** Fresh weight of transgenic grape suspension cells of EV and *hsfa2* mutant after 45°C for 80 min and 25°C for 7 d. Student’s *t*-test (^**^P < 0.01) was used to determine significant differences. ns: not significant.

**Figure 5:**
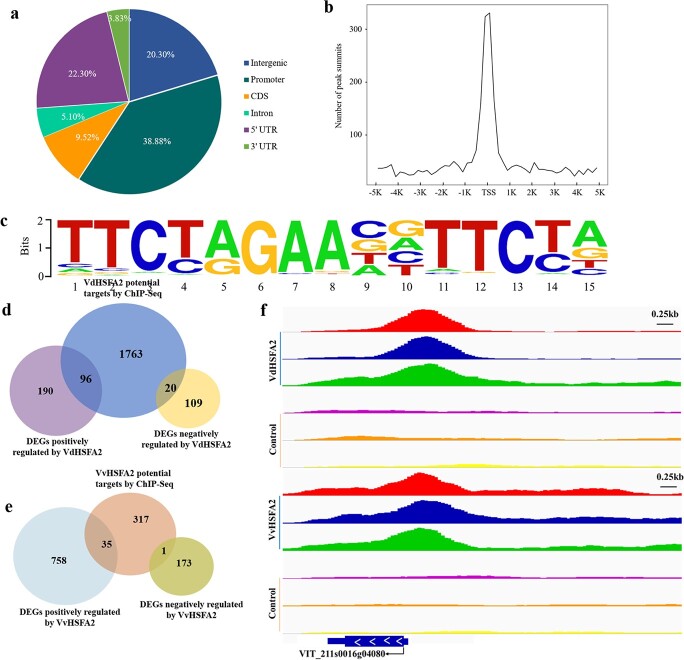
Genome-wide analysis of VdHSFA2 and VvHSFA2 targeted genes by ChIP-Seq and RNA-Seq. **a** Distribution of VdHSFA2 binding regions in three biological replicates in grape genome. **b** Distribution of VdHSFA2 binding sites around the transcription start sites (TSS) of genes. **a** and **b** of VvHSFA2 is shown in the Fig. S7 (see online supplementary material). **c** Motif of the most highly enriched VdHSFA2 and VvHSFA2 binding sites identified by Homer prediction. **d** Venn diagram showing an integration of ChIP-Seq and RNA-Seq results of VdHSFA2. **e** Venn diagram showing an integration of ChIP-Seq and RNA-Seq results of VvHSFA2. **f** Distribution of the VdHSFA2 and VvHSFA2 binding sites for MBF1c (VIT_211s0016g04080) gene loci. The binding sites were obtained from three independent biological replicates. The translation start site (ATG) positions and gene transcription direction were indicated with black arrows.

**Figure 6:**
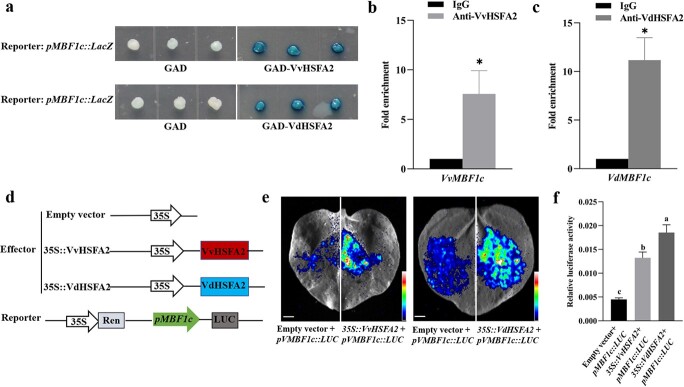
VvHSFA2 and VdHSFA2 positively regulate the expression of *MBF1c*. **a** Y1H assay of VvHSFA2 and VdHSFA2 binding to *MBF1c* promoter. The coding sequences of VdHSFA2 and VvHSFA2 were fused with GAD vector including Gal4 transcriptional activation domain (AD), respectively. Promoter of *MBF1c* was fused with LacZ reporter genes. Constructed vector were co-transformed into EGY48 yeast, the transformed yeast was grown on SD/−Trp/−Leu/–Ura/X-α-gal. The transformants with GAD and Reporter: *proMBF1c*::LacZ were used as negative control. **b** and **c** ChIP-qPCR verification of VvHSFA2 and VdHSFA2 binding to *MBF1c* promoter. A specific GFP antibody against VvHSFA2-GFP and VdHSFA2-GFP tag was used to precipitate chromatin. IgG was used as an antibody control. **d** Schematic representation of constructed plasmids used in the transient expression assays in *N. benthamiana*. A 574-bp promoter fragment of *MBF1c* that contains two HSE motifs was used to drive the expression of the firefly luciferase reporter gene. The internal control was Renilla luciferase gene driven by the 35S promoter. **e** and **f** Transactivation of the *MBF1c* promoter by VvHSFA2 and VdHSFA2 by luciferase activity assay. The representative images of an *N. benthamiana* leaves 48 h after infiltration are shown. The relative luciferase activity was measured. Data are based on three independent replicates. Duncan test (*P* < 0.05) or Student’s *t*-test (^*^P < 0.05) were used to determine significant difference.

To further address the function of *HSFA2* on thermotolerance in grape, we investigated the thermotolerance of grape suspension cells in which *HSFA2* was interrupted by CRISPR-Cas9. sgRNA is shown in Fig. 4a. The mutant hsfa2 grape suspension cells were detected by sequencing analysis. As shown in Fig. 4b, we took 10 colonies in which eight colonies were edited and resulted in five type mutations. The mutated amino acid sequences were shown in Fig. 4c, respectively. As expected, the appearance of *hsfa2* suspension cells was similar with empty vector grape suspension cells when they grew at 25°C (Fig. 4d). By contrast, *hsfa2* suspension cells became black after 45°C for 80 min and recovery at 25°C for 7 d (Fig. 4d). As shown in Fig. 4e, the fresh weight of controls and *hsfa2* cells declined significantly after heat treatment and recovery, but the fresh weight of *hsfa2* cells was lower than control cells. Collectively, these results indicated that *hsfa2* grape suspension cells were more sensitive to heat stress than control cells. In addition, we generated transiently transgenic *V. quinquangularis* plants by VdHSFA2 RNA interference (RNAi-HSFA2). The result showed that loss-function of *HSFA2* was more sensitive to heat stress than controls (Fig. S6, see online supplementary material).

**Figure 7:**
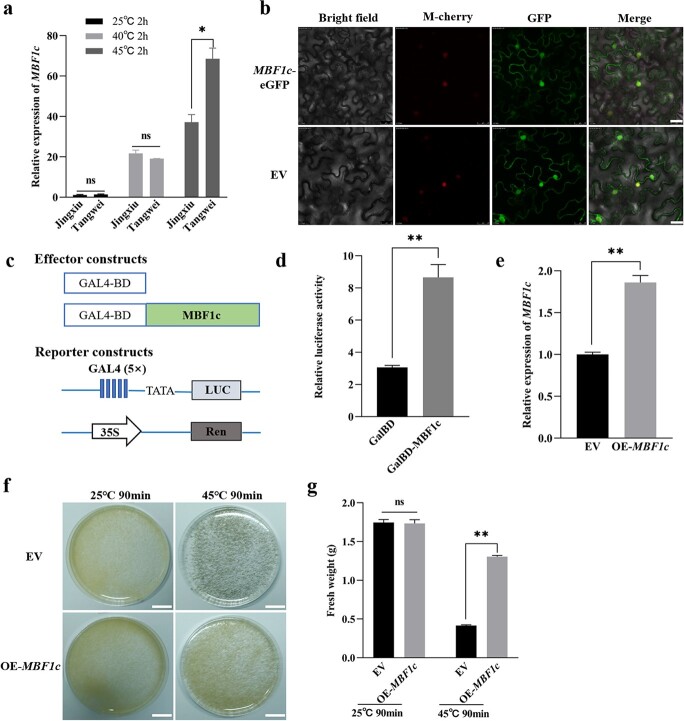
The expression, subcellular localization, transcriptional activities and function of MBF1c. **a** Expression analysis of *MBF1c* was conducted using RT-PCR. **b***MBF1c*-eGFP was transiently transformed into epidermal cells of *N. benthamiana* leaves, empty vector (EV) as control. Images under bright field (left), fluorescence (middle), and the merged images (right) are shown. Bar: 20 μm. **c** Schematic diagrams indicate constructed plasmids in the transient expression assays in Arabidopsis protoplasts. The CDS of *MBF1c* was cloned into CTB7-GAL4BD vector as the effector. Negative control was the empty vector. LUC was used as a reporter to detect the transcriptional activation of MBF1c. Ren was as internal reference. **d** Transcriptional activity analysis of MBF1c in *Arabidopsis* protoplasts by dual luciferase assay. Constructed vector was co-transformed Arabidopsis protoplasts with firefly LUC reporter vector and the renilla LUC vector. GalBD were used as negative control. **e** Expression analysis of *MBF1c* in transgenic grape suspension cells of empty vector (EV) and overexpressing *MBF1c* (OE-*MBF1c*) using RT-PCR. **f** Phenotypes of grape suspension cells of EV and OE*-MBF1c* after heat treatment and 25°C for 7 d. The suspension cells grown under 25°C were as control. Bar: 1.7 cm. **g** Fresh weight of transgenic grape suspension cells of EV and OE*-MBF1c* after heat treatment and 25°C for 7 d. Student’s *t*-test was used to determine significant difference (^*^P < 0.05; ^**^P < 0.01). ns: not significant.

### Potential genes targeted by VvHSFA2 and VdHSFA2

We performed a ChIP-Seq assay in order to investigate the target genes of VvHSFA2 and VdHSFA2. We pulled down the putative VvHSFA2-bound and VdHSFA2-bound DNA sequences using antibodies specific for the VvHSFA2-GFP and VdHSFA2-GFP tags based on three biological replicates. Model Based Analysis of ChIP-Seq (MACS) was used to predict VvHSFA2 and VdHSFA2 binding sites, setting a false discovery cut-off of 0.05. The total number of reads and mapped reads of ChIP-Seq are shown in Table S3 (see online supplementary material). The total number of reads were from 34 183 564 to 49 015 034, and mapped reads were from 18 383 583 to 32 683 141. Their mapping rates were up to 47.41%–68.81% according to default parameters by BWA software (version: 0.7.15-r1140) [45]. Annotation of the VdHSFA2 binding peak regions showed that 38.8% were mapped in promoter regions (Fig. 5a and b).

**Figure 8:**
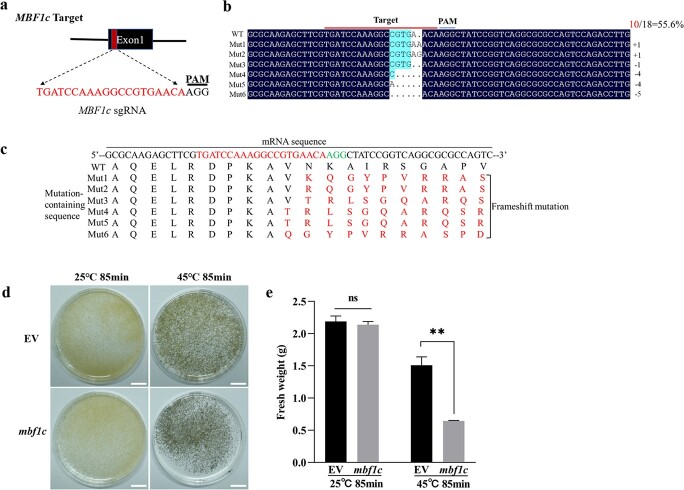
**.** Knockout of *MBF1c* decreased heat tolerance in grape suspension cells. **a** Diagram illustration of *MBF1c* target sites design using CRISPR/Cas9 technology. The sequences of sgRNA are shown in red, PAM are in black. **b** Identification of *MBF1c* knockout mutant (mbf1c). The total of 18 colonies for each target were analysed. Red represents the number of mutated sequences. Sequencing results on both sides of the target site are shown. The wild type (WT) or mutant (mut) lines are shown on the left. Mutant types are shown on the right. **c** Mutations of amino acids in corresponding mutant sequencing in **b**. **d** Phenotypes of grape suspension cells of EV and *mbf1c* mutant after heat treatments (45°C for 85 min) and 25°C for 7 d. The grape suspension cells grown under 25°C were as control. Bar: 1.5 cm. **e** Fresh weight of transgenic grape suspension cells of EV and *mbf1c* mutant after 45°C for 85 min and 25°C for 7 d. Student’s *t*-test was used to determine significant difference (^**^P < 0.01). ns: not significant.

For VvHSFA2 peaks, 23.51% were located at promoter regions (Fig. S7a and b, see online supplementary material). The DNA motif bound by HSFA2 within peak sequences were identified with the Homer software. Despite the most significantly enriched motif of both VdHSFA2 and VvHSFA2 being Motif 1 (core sequence TTCNNGAANNTTCNN) (Fig. 5c), the motif analysis reported 108 motifs for VdHSFA2 (Table S4, see online supplementary material), while only 20 motifs of VvHSFA2 (Table S5, see online supplementary material). The identified 2040 peaks corresponded to 1879 genes in VdHSFA2 ChIP-Seq assay, whereas 621 peaks fitted to 353 genes in VvHSFA2 ChIP-Seq assay (Fig. 5d and e; Tables S6 and S7, see online supplementary material).

To determine which were transcriptionally regulated by HSFA2 among the above bounding genes, we performed RNA-Seq analysis of EV, OE-*VvHSFA2*, and OE-*VdHSFA2* grape suspension cells. The total number of reads and mapped reads of RNA-Seq are shown in Table S8 (see online supplementary material). The total number of reads were from 50 236 622 to 64 994 682, and mapped reads were from 46 101 972 to 58 898 181. Their mapping rates were up to 88.67%–91.77% based on default parameters by Hisat2 software (http://ccb.jhu.edu/software/hisat2/index.shtml) [46]. Different expressed genes (DEGs) in OE-*VvHSFA2* and OE-*VdHSFA2* grape suspension cells are shown in Tables S9 and S10 (see online supplementary material), respectively. The combination of ChIP-Seq and RNA-Seq data showed that VdHSFA2 may directly regulate 116 genes among which 96 were up-regulated and 20 were down-regulated (Fig. 5d; Table S11, see online supplementary material); VvHSFA2 may directly regulate 36 genes among which 35 were up-regulated and one was down-regulated (Fig. 5e; Table S12, see online supplementary material). A total of 116 VdHSFA2-regulated genes and 36 VvHSFA2-regulated genes were characterized based on the Gene Ontology (GO) database Goatools (https://github.com/tanghaibao/Goatools), respectively. Among the categories, the GO terms such as response to heat, response to hydrogen peroxide, response to temperature stimulus, cellular response to heat, and UDP-galactosyltransferase activity were both enriched in VdHSFA2-regulated genes and VvHSFA2-regulated genes, but the numbers in these GO terms were higher in VdHSFA2-regulated genes than in VvHSFA2-regulated genes (Fig. S7c and d; Tables S13 and S14, see online supplementary material).

Half of the genes directly regulated by VvHSFA2 are 18 HSPs, including many HSP20, but also some HSP40, HSP70, HSP90, and HSP100. Additional regulated genes related to heat stress responses include MBF1c, HSFB2a, and universal stress protein PHO34. Galactinol synthase1 (GOLS1), which is involved in the synthesis of compatible solutes from the raffinose family oligosaccharides (RFOs), appeared regulated by VvHSFA2, as well as a catalase, a GEM-like protein, a BAG domain protein, a E3 ubiquitin-protein ligase (Table S12, see online supplementary material).

Most of the genes suspected to be directly regulated by VvHSFA2 were also extracted from the data collected with VdHSFA2. The genes directly regulated by VdHSFA2 included 38 HSPs family members, VdHSFA2 itself, various enzymes from the RFOs synthetic pathway, different transcription factors from ERF, MYB, WRKY, and bZIP families, two UDP-glycosyltransferase, and a betaine aldehyde dehydrogenase 1 (Table S11, see online supplementary material). The common and specific genes directly regulated by VdHSFA2 and/or VvHSFA2 were listed in Tables S15, S16, and S17, respectively (see online supplementary material).

### HSFA2 mediates thermotolerance in grapevine through regulating *MBF1c* expression

MBF1c is a transcriptional coactivator with high conservation and plays important roles in diverse processes including thermotolerance [25, 31]. In our study, *MBF1c* belongs to the list of VdHSFA2-regulated genes and VvHSFA2-regulated genes (Fig. 5f; Tables S11 and S12, see online supplementary material). To evaluate if HSFA2 can regulate *MBF1c* expression and to explore if there is any difference in regulation efficiency between VdHSFA2 and VvHSFA2 for *MBF1c*, yeast one-hybrid assay (Y1H), ChIP-qPCR and luciferase (LUC) reporter assay were performed. Firstly, we cloned and sequenced the promoter regions from ‘Jingxiu’ and ‘Tangwei’, respectively. We found that they have the same promoter sequences (Fig. S8, see online supplementary material). According to the ChIP peak of VvHSFA2 and VdHSFA2 binding *MBF1c* promoter (Fig. 5f), we chose to clone the core 574 bp fragment of *MBF1c* promoter into the pLacZ reporter vector. As shown in Fig. 6a, VvHSFA2 and VdHSFA2 can strongly activate the LacZ reporter gene expression, indicating that VvHSFA2 and VdHSFA2 can directly bind to *MBF1c*. This was confirmed by ChIP-qPCR, our results indicating that both VvHSFA2 and VdHSFA2 can directly bind to the *MBF1c* promoter (Fig. 6b and c). To detect if the VvHSFA2 and VdHSFA2 can induce the expression of *MBF1c in planta*, luciferase (LUC) reporter assay was conducted using *N. benthamiana* leaves transiently transformed with 35S::VvHSFA2 and 35S::VdHSFA2 plasmids used as effectors and *proMBF1c::LUC* plasmid as reporter. When empty vector was used, weak LUC activity was observed, whereas overexpression of VvHSFA2 or VdHSFA2 enhanced significantly the LUC activity driven by the *MBF1c* promoter. Again, VdHSFA2 can transactivate the pro*MBF1c*-dependent LUC activity in a stronger manner when compared to the effect of VvHSFA2 (Fig. 6e and f). Taken together, VvHSFA2 and VdHSFA2 could directly bind to the promoter of *MBF1c in vitro* and *in vivo*, in addition, the transcriptional activation ability of VdHSFA2 to *MBF1c* was stronger than VvHSFA2.

In addition, we explored whether MBF1c can directly regulate *HSFA2*. As a result, MBF1c can’t bind to *VvHSFA2* and *VdHSFA2* promoters by Y1H (Fig. S10, see online supplementary material). At the same time, we found that MBF1c cannott interact physically with VvHSFA2 or VdHSFA2 (Fig. S11, see online supplementary material).

### Identification of MBF1c characteristics and verification of MBF1c-induced heat tolerance in grape

Accumulation of *MBF1c* transcripts was determined in ‘Jingxiu’ and ‘Tangwei’ grapevines exposed to HS. The same leaf samples as the *VvHSFA2* and *VdHSFA2* analysis was used to analyse *MBF1c* expression by qRT-PCR. As shown in Fig. 7a, expression of *MBF1c* increased in ‘Jingxiu’ and ‘Tangwei’ leaves exposed to HS. The *MBF1c* CDS of ‘Jingxiu’ and ‘Tangwei’ were cloned and sequenced, revealing differences for two nucleotides without consequence for the coded amino acid sequence (Fig. S9, see online supplementary material). We further explored the subcellular localization of MBF1c using MBF1c-GFP recombinant protein transiently expressed into *N. benthamiana* leaves. Fluorescence analysis revealed that *MBF1c*-GFP was detected in both cytosol and nuclei (Fig. 7b). The transcriptional activity of MBF1c assay was demonstrated in *Arabidopsis* protoplasts, as described above when studying transcriptional activity of HSFA2. The effector and reporter constructs are shown in Fig. 7c. The results showed that MBF1c possesses transcriptional activity (Fig. 7d).

To explore the role of *MBF1c* in grapevine thermotolerance, firstly, the recombinant protein *MBF1c*-GFP was stably expressed in grape suspension cells (OE-*MBF1c*). Compared with control cells (EV), *MBF1c* expression was ~2 fold higher in OE-*MBF1c* cells (Fig. 7e). The appearance of OE-*MBF1c* suspension cells was similar with EV grape suspension cells when they grew at 25°C (Fig. 7f). However, more OE-*MBF1c* suspension cells were still well than EV suspension cells after heat treatment of 45°C for 90 min and recovery 7 d at 25°C (Fig. 7f). As shown in Fig. 7g, the fresh weight of controls and OE-*MBF1c* suspension cells declined significantly after heat treatment and recovery, but the fresh weight of OE-*MBF1c* suspension cells was higher than EV cells. Secondly, we generated MBF1c mutant cells (*mbf1c*) of grape suspension cells in which *MBF1c* was interrupted by CRISPR-Cas9 gene editing. sgRNA is shown in Fig. 8a.

The mutant MBF1c (*mbf1c*) of grape suspension cells were detected by sequencing analysis. As shown in Fig. 8b, we took 18 colonies in which 10 colonies were edited and resulted in six type mutations. The mutated amino acid sequences were shown in Fig. 8c, respectively. Sequencing analysis revealed that *MBF1c* was successfully edited (Fig. 8b and c). As expected, the appearance of *mbf1c* grape suspension cells was similar with EV cell when grown at 25°C (Fig. 8d). However, more *mbf1c* suspension cells became black than EV cells after heat treatment of 45°C for 85 min and recovery 7 d at 25°C (Fig. 8d). At the same time, the fresh weight of controls and *mbf1c* cells declined significantly, but the fresh weight of *mbf1c* cells was lower than EV cells (Fig. 8e). Taken together, the results suggests that *MBF1c* plays an important role in thermotolerance of grape.

## Discussion

In many model plants or common crops, such as Arabidopsis, rice, maize, wheat, tomato, soybean, HSFs play an important role in plant thermotolerance [15, 17, 47–50], acting as the core regulatory factors for HSR [4, 8, 9]. HSFA2 in particular activates the expression of HS-regulated genes and is important for thermotolerance acquisition and the heat stress memory [16, 51]. In this context, grapevine responses to heat stress are particularly important but still far from being fully understood [52, 53]. A similar pivotal role was proposed for HSFA2 in grapevine [5, 10, 19] but its precise function remains to be determined. Most accessions of *V. vinifera,* among which are the main consumed grape, have relatively weaker heat tolerance than wild *V. daviddi* and *V. quinquangularis* originated from the heat regions of southern China [41]. Consequently, the identification of HSFA2 sequence variation between these grape accessions would be of particular interest to help future breeding strategies aiming at producing grapevine with high quality and yield in warmer climates. To the best of our knowledge, there is only a report that a variation in the intron of HSFA2 attributes to enhanced thermotolerance in tomato [9], and no reports linking different HSFA2 variation to grapevine or the other plants.

In our previous studies, the expression of *HSFA2* in *V. vinifera* leaves and fruits gradually increased with elevating temperature [5, 19]. Therefore, we firstly investigated *HSFA2* expression change in *V. vinifera* ‘Jingxiu’ and *Vitis daviddi* ‘Tangwei’ grapevines exposed to high temperatures. As shown in Fig. 1a, the transcription levels of *HSFA2* were higher in leaves of ‘Tangwei’ than ‘Jingxiu’ after similar heat stress. Accordingly, we also observed that the promoter activity of *VdHSFA2* from ‘Tangwei’ was higher than *VvHSFA2* from ‘Jingxiu’ under 25°C or 37°C (Fig. 1b, c and d). This result may be related to the stronger thermotolerance of *V. daviddi* when compared to *V. vinifera*. However, importantly, we want to know if natural variations of HSFA2 coding region confer thermotolerance in grapevine. The present work indicated that VvHSFA2 and VdHSFA2 are both located into the nucleus, while VdHSFA2 has higher transcriptional activity than VvHSFA2 (Fig. 2). Interestingly, *VvHSFA2* and *VdHSFA2* have 11 single nucleotide polymorphisms in the coding region (Fig. S3, see online supplementary material) leading to differences for seven amino acid residues distributed in DBD, HR-A/B, and AHA domains (Fig. 2a). Among these, although three amino acid residues difference between VvHSFA2 and VdHSFA2 were located at their AHA1 and adjacent domain (Fig. 2a), only when Thr^315^ in the AHA1 domain for VdHSFA2 was change into Ile^315^, VdHSFA2 transcriptional activity was decreased (Fig. 2f and g). The results indicate that Thr^315^ may be essential functional amino acid for the higher transcriptional ability of HSFA2. For instance, Arabidopsis HSFA2 activity was regulated through its Thr^249^ phosphorylation upon HS [54]. As mentioned above, our knowledge about the relationship between natural variations in key genes and plant thermotolerance efficiency is still fragmentary, particularly in horticulture crops. Recently, Li *et al.* found that an amino acid change between African rice and Asian rice leads to proteasome activity difference of *thermo-tolerance 1* (*TT1*) [55]. It was shown that natural variation of *SLG1* (cytosolic tRNA 2-thiolation protein 2) enhances higher heat tolerance in *Oryza sativa* subsp. *indica* compared to *japonica* [56]. There was also a report about natural variation about HSFA2 in tomato, but this research showed the SNP in HSFA2 intron brought about the different HSFA2 transcription splicing efficiency causing changes in thermotolerance, rather than SNPs of HSFA2 promoter or coding sequences [9]. To gain insights into whether the variation of HSFA2 is associated with grape thermotolerance, we evaluated the heat tolerance of various *Vitis*. Thirty-eight *V. vinifera* accessions bearing the same HSFA2 coding region (VvHSFA2) were described as heat sensitive, while two *V. davidii* accessions and one *V. quinquangularis* accession with the same HSFA2 coding region (VdHSFA2) were better tolerant to heat (Table 1; Table S1, see online supplementary material). In addition, some *Vitis amurensis* was also reported as relative thermotolerant [42]. However, we found that VaHSFA2 from *V. amurensis* possesses Ile^315^ (data not shown), which is the same as VvHSFA2. *V. daviddi* and *V. quinquangularis* are wild species distributed in the south of China. However, *V. amurensis* distributes in the northeast of China. Therefore, based on their different geographical distribution, we speculate that their thermotolerance mechanism may also be different; the thermotolerance of *V. amurensis* may not be determined by HSFA2 but by other factors. For example, a few reports demonstrated that DREB/CBF-type transcription factors function in cold stress-response as well as heat and drought response in plants [57, 58]. Therefore, as a cold tolerant grape, *V. amurensis* maybe acquire higher thermotolerance by DREB/CBF-type transcription factors.

So far, no genetic evidence was reported about the effect of HSFA2 on grape thermotolerance. Our work revealed that overexpression and mutation of *HSFA2* increased or decreased thermotolerance of grape suspension cells, respectively (Figs 3 and 4). Although *VdHSFA2* showed lower expression level than *VvHSFA2* in the corresponding overexpression lines of grape suspension cells, OE-*VdHSFA2* cells still presented higher thermotolerance capacity than OE-*VvHSFA2* cells (Fig. 3d). These results should be the consequence of a higher transcriptional activity of *VdHSFA2* when compared to *VvHSFA2*. To discard the influence of different overexpression levels of *VdHSFA2* and *VvHSFA2* on target genes, we generated tobacco leaves with the same expression of *VdHSFA2* and *VvHSFA2*, and found that expression levels of *HSP22.0* (one HSFA2 target gene) in OE-*VdHSFA2* cells were higher than OE-*VvHSFA2* lines (Fig. 3e and f). These results confirmed that transcriptional activity of *VdHSFA2* is higher than *VvHSFA2*.

Because *VdHSFA2* was more efficient than *VvHSFA2* in improving grape heat tolerance (Fig. 3), we looked for potential difference in targeted genes between VvHSFA2 and VdHSFA2. In recent years, integration of ChIP-Seq and RNA-Seq has been an effective strategy for exploring targeted genes of transcriptional factor. Li *et al.* reported transcriptional regulatory framework of Opaque2 in *Zea mays* by integration of ChIP-Seq and RNA-Seq [59]. Zhang *et al.* (2020) combined ChIP-Seq and RNA-Seq to point out IbBBX24 binding to IbJAZ10 and IbMYC2 [60]. Here, we reported that VdHSFA2 can directly regulated 116 genes while VvHSFA2 can directly regulated only 36 genes after data mining of our ChIP-Seq and RNA-Seq experiments (Fig. 5d and e). Among these target genes, we found various *HSPs* and *GOLSs* already reported to be transactivated by HSFA2 in both Arabidopsis and grape [17, 19]. Interestingly, we noticed that more heat stress related genes, mostly from the HSP family were regulated by VdHSFA2 than VvHSFA2 (Tables S11, S12, S13, and S14, see online supplementary material).

Rienth *et al.* reported that a very strong correlation remained in the expression of *HSFA2* and *MBF1c* in *V. vinifera* berries, following 2 hours of a rather moderate thermal stress (37°C) at the whole plant level, at different developmental stages and times during a nychthemeral cycle [33]. The result indicated that there may be a regulation relationship between *HSFA2* and *MBF1c* in grape. Based on our results of ChIP-Seq and RNA-Seq, MBF1c was found to be a putative target gene of HSFA2 (Fig. 5f; Tables S11 and S12, see online supplementary material). Combining yeast one hybrid, ChIP-qPCR and luciferase (LUC) reporter assay, our results showed that HSFA2 can bind to the *MBF1c* promoter and transactivate its expression (Fig. 6). However, MBF1c can’t bind to the *HSFA2* promoter (Fig. S10, see online supplementary material). Previous study described MBF1c as a bridge factor [20]; however, our results indicated that MBF1c can’t interact physically with HSFA2 (Fig. S11, see online supplementary material). Although the roles of MBF1c on heat, drought and salt tolerance were reported in some species, such as Arabidopsis, tomato, tobacco, potato, pepper, and wheat [24, 29, 61–63], the function of MBF1c in grape has not been studied yet. *MBF1c* transcripts accumulated in grape leaves exposed to heat stress (Fig. 7a). In our research, overexpression and mutation of MBF1c in grape suspension cells resulted in higher and weaker thermotolerance, respectively (Figs 7 and 8). These data indicate that MBF1c induces grape thermotolerance.

In conclusion, our work revealed that grape thermotolerance was modulated by HSFA2 variations, with the VdHSFA2 conferring higher heat tolerance than the VvHSFA2. The transcriptional activation activities of *VdHSFA2* are higher than the ones from *VvHSFA2*, and VdHSFA2 can regulate more target genes than VvHSFA2. Interestingly, we found that the variation of single amino acid (Thr315Ile) only in the AHA1 motif results in difference of transcriptional activities between VdHSFA2 and VvHSFA2. This variation discovery could be of potential significance for understanding plant thermotolerance and for supporting future molecular breeding of thermotolerant grapevine. Finally, MBF1c is a target gene of HSFA2 and plays a relevant role in the grape response to heat stress.

## Materials and methods

### Plant materials and heat treatments

One-year-old ‘Jingxiu’ (*V. vinifera*) and ‘Tangwei’ (*V. davidii*) grapevines were used in this study. The grapevine growth condition and heat treatments were carried out as described by Jiang *et al.* [5]. The grapevines were grown in greenhouse with the relative humidity 70%–80%, temperature 25°C/18°C day/night, and light 800 μmol m^−2^ s^−1^. When the sixth leaves (counting from up to down) were mature, grapevines were divided into three groups, and each group had three seedlings at least. Grapevines of each group were treated at 25°C, 40°C, and 45°C for 2 h (all conditions were the same as above their growth conditions except for temperature), respectively. A total of 41 grape accessions were used in this study (Table S1, see online supplementary material). The grape accessions were grown at the Germplasm Repository for Grapevines in the Institute of Botany of the Chinese Academy of Sciences, located in Beijing. Healthy 30-day leaves were used to evaluate the thermotolerance. The evaluation of these germplasms was conducted in May of 2019 according to Xu *et al.* [41]. Tissue culture grape plants ‘Jingxiu’ (*V. vinifera*) and ‘Summer Black’ (*V. vinifera* × *Vitis labrusca*) were used for transiently overexpression of VdHSFA2 and VvHSFA2, among which ‘Summer Black’ was heat sensitive and had the same HSFA2 sequence as ‘Jingxiu’ (*V. vinifera*). One-year-old *V. quinquangularis* plants were used for transiently RNA interference of VdHSFA2. *A. thaliana* ecotype Columbia-0 (Col-0) and *Nicotiana benthamiana* were grown in a growth chamber with their suitable growing conditions.

*A. thaliana* ecotype Columbia-0 (Col-0) and *N. benthamiana* were grown in the greenhouse with conditions of a 16 h/8 h, light/dark cycle, and the relative humidity and temperature was 70–75%, 23°C, respectively. These materials were used for the research of *HSFA2* or *MBF1c* function.

### VvHSFA2 and VdHSFA2 gene isolation and analysis

Total RNA was extracted from mature grape leaves using RNAprep Pure PlantKit (Tiangen, Beijing, China). cDNA syntheses were performed using total RNA and the HiScript® III 1st Strand cDNA Synthesis Kit (Vazyme, Nanjing, China). Based on the gene sequence of Pinot Noir HSFA2 acquired from the grape Genome Browser (https://phytozome.jgi. doe.gov/pz/portal.html), the primer pair for HSFA2 was designed using DNAMAN. VvHSFA2 and VdHSFA2 were cloned from cDNA by PCR. The HSFA2 sequences were aligned using DNAMAN. The domain was analysed according to *Arabidopsis* and *O. sativa* HSFs family. The primers used in the present study are listed in Table S2 (see online supplementary material).

### Gene expression analysis

AceQ qPCR SYBR Green Master Mix (Vazyme, Nanjing, China) was used to conduct qRT-PCR. *VvActin* was used as an internal control. The qPCR condition was carried out according to the manufacturer’s instructions. The gene relative expression level was calculated using the 2^-ΔΔCT^ method.

### Analysis of *HSFA2* promoter activity

The isolated 2 kb fragments of the *VvHSFA2* and *VdHSFA2* promoters were fused to a pGreenII-0800-LUC vector, respectively. CaMV35S::Ren was used as an internal control. The constructed vector was transformed into Arabidopsis protoplast. The transformed protoplasts were put at 25°C for 16 h before harvest. In addition, the constructed vector was transformed into Agrobacterium GV3101 (pSoup) for transformation of *N. benthamiana* leaves. After 48 h, some transformed lines were treated at 37°C for 1 h or 2 h, and some allowed to remain in the growth chamber (25°C). The activity of firefly luciferase (LUC) and Renilla luciferase (REN) were measured by Dual-Luciferase Reporter Assay System kit (Promega, Madison, USA). The Tanon 5200 Multi luminometer (China) was used to detect the fluorescence signal.

### Subcellular localization

The vector 2300-GFP was used to study the subcellular localization of VvHSFA2 and VdHSFA2. The coding sequence (CDS) of *VvHSFA2* was fused with 2300-GFP vector and signed as *VvHSFA2*-eGFP. The *VdHSFA2*-eGFP was constructed in the same way. The empty GFP vector (EV) was used as control. *VvHSFA2*-eGFP, *VdHSFA2*-eGFP, and EV were transformed into Agrobacterium EHA105, respectively. *N. benthamiana* leaves were injected with transformed Agrobacterium for transient transformation. The Agrobacterium transformed H2B-mCherry was used as nuclei marker. Leica TCS SP5 Confocal Scanning Microscope was used to detect the transformed *N. benthamiana* fluorescence signal after 48–72 h incubation period.

### Transcriptional activity assay

Transcriptional activity assay was firstly conducted in yeast. The CDS of VvHSFA2 and VdHSFA2 were fused to the GAL4 BD vector, and the constructed vector was named as BD-VvHSFA2, BD-VdHSFA2, respectively. The constructed plasmids were transformed to yeast strain Y2HGold. Positive and negative controls were the yeast clones containing combinations of AD-T with BD-p53 and BD-Lam, respectively. Transcriptional activation activity was evaluated by spot assay and alpha-galactosidase activity.

Transcriptional activity assay was also conducted in *Arabidopsis* protoplasts by dual luciferase reporter assay. The CDS of *VvHSFA2*, *VdHSFA2*, *VdHSFA2* mutation, and *MBF1c* were cloned into CTB7-GAL4BD vector as the effector, signed as GalBD-VvHSFA2, GalBD-VdHSFA2, GalBD-VdHSFA2mut1/2/3, and GalBD-MBF1c, respectively. The empty vector was used as negative control. LUC was used as reporter to detect the transcriptional activation of VvHSFA2, VdHSFA2, VdHSFA2 mutation, and MBF1c. Ren was the internal reference. The reporter and effector plasmids were co-transformed into Arabidopsis protoplasts by PEG-mediated transformation. The transformed Arabidopsis protoplasts were cultured at 25°C in the dark overnight. Dual-luciferase activity was measured by the Dual-Luciferase Reporter Assay System kit (Promega, Madison, USA).

### Stable transformation of VvHSFA2 and VdHSFA2 in grape and heat treatments

Grape transformations were made in ‘41B’ (*V. vinifera* × *Vitis berlandieri*) grape suspension cells as previous described [64]. Overexpressed *VvHSFA2* (OE-*VvHSFA2*) and *VdHSFA2* (OE-*VdHSFA2*) vectors were constructed, respectively. Empty vector (EV) was as control. CRISPR/Cas9-based gene editing in grape was conducted according to the method of Ma and Liu [65]. The verified sequence was fused into sgRNA according to the online tool CRISPR-GE (Genome Editing)-Liu YG Lab (scau.edu.cn) [65]. sgRNA intermediate vectors with Arabidopsis U3b promoter (PYLsgRNA-AtU3b) was used in this study. HSFA2 and MBF1c target were imported into PYLsgRNA-AtU3b, respectively. The designed sgRNAs were ligated with pYLCRISPR/Cas9P35S-N by homologous recombination. The constructed vectors were transformed into Agrobacterium EHA105, then the transformed Agrobacterium were co-cultured with grape suspension cells. The infected grape suspension cells were cultured in liquid GM medium supplemented with antibiotic. The successfully transformed overexpression lines were identified by qRT-PCR and fluorescence detection. The successfully transformed mutation lines were identified by sequencing.

Grape suspension cells were grown at 25°C for six days, then they were treated as the following treatments: (i) the samples of control group remained at the optimal conditions (120 rpm, 25°C); and (ii) the samples of the heat treatment groups were transferred into shaking incubator (120 rpm, 45°C). When the heat treatments were finished, grape suspension cells were recovered at 25°C for seven days. The heat treatments time was identified based on the survival ability (Fresh weight) at 7 d after recovery (120 rpm, 25°C) from different heat treatments, the data is shown in Fig. S5 (see online supplementary material). Grape suspension cells were taken out from medium, and dried with blotting paper. Then these cells were weighted by electronic scales (Mettler Toledo, Shanghai, China).

### Transient transformation of VvHSFA2 and VdHSFA2 in grapevine and heat treatments

Agrobacterium EHA105 that transformed OE-*VvHSFA2* or OE-VdHSFA2 vectors were used for overexpression experiments, respectively. Agrobacterium EHA105 transformed empty vector was used as control. The HSFA2-specific sequence and its specific reverse transcription sequence were cloned into pFGC5941 through homologous recombination to obtain a functionally deficient HSFA2 construct, generating small interfering RNA (siRNA). The constructed vectors were transformed into Agrobacterium EHA105.

Six-week-old tissue culture grape plantlets of ‘Jingxiu’ and ‘Summer Black’ were infiltrated by Agrobacterium EHA105 with the above overexpressing construct. After infiltration, the plantlets were transferred to tissue culture medium for 4 days. Then, the plants were treated by high temperature: (i) the control samples remained at the optimal conditions (25°C); and (ii) the samples of the heat treatment groups were treated at 45°C 3 h for ‘Jingxiu’ and 45°C 2 h for ‘Summer Black’. Four weeks after one-year-old tissue culture plants of *V. quinquangularis* germinated, these plants were infiltrated by Agrobacterium EHA105 with the above silencing construct. Then, the infiltrated plants were transferred to soil. After 4 days, the infiltrated plants were treated by high temperature: (i) the control plants remained at the optimal conditions (25°C); and (ii) the heat treatment group plants were treated for 150 min at 40°C. The relative electrolyte leakage was measured as follows: 10 leaves discs (0.5 cm in diameter) were incubated in 5 ml of distilled water, then conductivity before and after being boiled was measured using industrial conductivity [66].

### ChIP-Seq analysis

Overexpressed *VvHSFA2*, *VdHSFA2* and empty lines in grape suspension cells were sampled for the ChIP-Seq analysis. Every sample with three biological replicates. The ChIP assay was conducted according Landt *et al.* [67], with some modifications. In brief, 3–5 g samples were used to cross-link DNA and protein with 1% (v/v) formaldehyde, and these samples were ground into powder in liquid nitrogen. Purified DNA-protein complexes were sonicated and incubated with anti-GFP antibody (Abeam, ab290). The precipitated DNA was purified, dissolved in distilled water, and stored at −80°C to construct an Illumina sequencing library. ChIP-Seq was conducted by Igenebook (Wuhan, China). The reads from ChIP-Seq were aligned with the genome of *V. vinifera* from the grapevine database (http://genomes.cribi.unipd.it/DATA/V2/V2/). The mapped reads were applied to MACS software (version: 2.1.1.20160309) to identify regions potentially bound by VvHSFA2 and VdHSFA2. The DNA motif within peak sequences was identified with Homer software (version 3).

### RNA-Seq analysis

For RNA-Seq, overexpressed VvHSFA2, VdHSFA2, and empty lines in grape suspension cells were sampled. RNAprep Pure PlantKit (Tiangen, Beijing, China) was used to extract total RNA. RNA quality was checked with the NanoDrop2000, agarose gel electrophoresis and Agilent 2100. According to the Illumina standard instructions, RNA-Seq libraries were prepared. RNA-Seq was implemented on the Illumina HiSeq 6000 platform by Shanghai Majorbio. DEGs were defined as those with *P*-value (*P* ≤ 0.05) and the fold change (|log2FC| ≥ 1).

### ChIP-qPCR

The prepared DNA in ChIP was used for qPCR. The fold changes were calculated based on the relative enrichment in anti-GFP compared with anti-IgG immunoprecipitate. The expression levels were normalized to the input sample. Each sample was evaluated based on three replicates.

### Yeast one-hybrid assay

Yeast one-hybrid assay was conducted as previously described Lin *et al.* [68]. The CDS of *VvHSFA2* and *VdHSFA2* were constructed into GAD vector (contain GAL4 transcriptional activation domain), respectively. The successfully constructed vector were signed as GAD-VvHSFA2 and GAD-VdHSFA2, respectively. The *MBF1c* promoter fragment contained HSE motif and was constructed into pLacZ vector, signed as *proMBF1c::LacZ*. Plasmids for AD fusions were each co-transformed with LacZ reporter constructs into yeast strain EGY48. Transformed yeast was grown on SD/−Trp/-Ura dropout plates containing X-gal for blue color reaction.

### Dual-luciferase assay

To explore the effect of HSFA2 on MBF1c transcriptional regulation, we performed LUC reporter assays in *N. benthamiana* leaves. The *MBF1c* core promoter containing the HSFA2 binding motif was constructed into pGreen0800-LUC to generate the reporter vector, *proMBF1c::LUC*. The *35S::VvHSFA2* and *35S::VdHSFA2* vectors were used as effectors, and the 35S empty vector was used as a control. The constructed plasmids were transformed into Agrobacterium EHA105, and *N. benthamiana* leaves were co-infected by Agrobacterium infiltration as Wang *et al.* [64] described. LUC activity was detected using Dual-Luciferase Reporter Assay System kit (Promega, Madison, USA). The analysis was performed in at least three biological replicates.

### Yeast two-hybrid assays

Yeast two-hybrid (Y2H) was performed using the Matchmaker Gold yeast two-hybrid system (Clontech, Palo Alto, USA). The CDS of HSFA2 and MBF1c were constructed into pGADT7 and pGBKT7 vectors, respectively. The yeast strain Y2HGold was co-transformed with different combinations of pGADT7 and pGBKT7 recombinant vectors and cultured on SD/−Leu/−Trp selection medium. Positive colonies were plated on SD/−Leu/−Trp/-His/−Ade selection medium, providing 40 mg L of 1x-α-Gal. The positive control was AD-T combined with BD-p53, and the negative control was AD-T combined BD-Lam.

## Supplementary Material

Web_Material_uhac250

## Data Availability

The data used to support the findings of this study are available from the corresponding authors upon reasonable request.
