# Supplementary material for: Natural variations of HSFA2 enhance thermotolerance in grapevine
Source: Hortic Res. 2022 Nov 10;10(1):uhac250. doi: 10.1093/hr/uhac250 (PMC9832954; doi:10.1093/hr/uhac250)
Supplement: Web_Material_uhac250 [file web_material_uhac250.zip › 20221022-Revised Supplementary Figures S1-S11.docx]

**Supplementary Fig. S1 Sequence comparison of *HSFA2* promoters in *Vitis vinifera* ‘Jingxiu’ and *Vitis davidii* ‘Tangwei’.**

The promoter sequence was cloned 2kb upstream from transcription start site. Alignment was performed using DNAMAN.


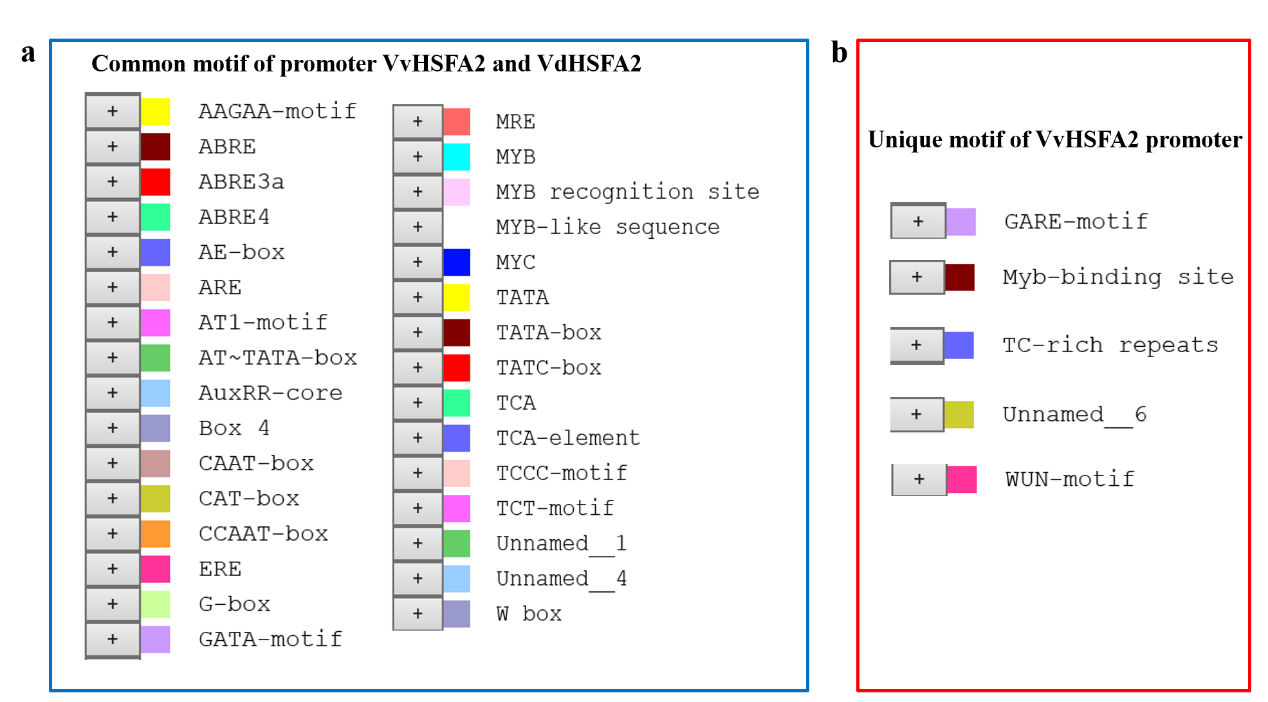


**Supplementary Fig. S2 Comparison of promoter motifs between *VvHSFA2* and *VdHSFA2*.**

The Motif analysis of *VvHSFA2* and *VdHSFA2* promoters by Plant CARE. **a** The common motifs between *VvHSFA2* and *VdHSFA2* promoters. **b** The unique motifs of *VvHSFA2* promoters

**Supplementary Fig. S3 Comparison of *HSFA2* coding sequence of *Vitis vinifera* ‘Jingxiu’ and *Vitis davidii* ‘Tangwei’.**

Alignment was performed using DNAMAN.

**
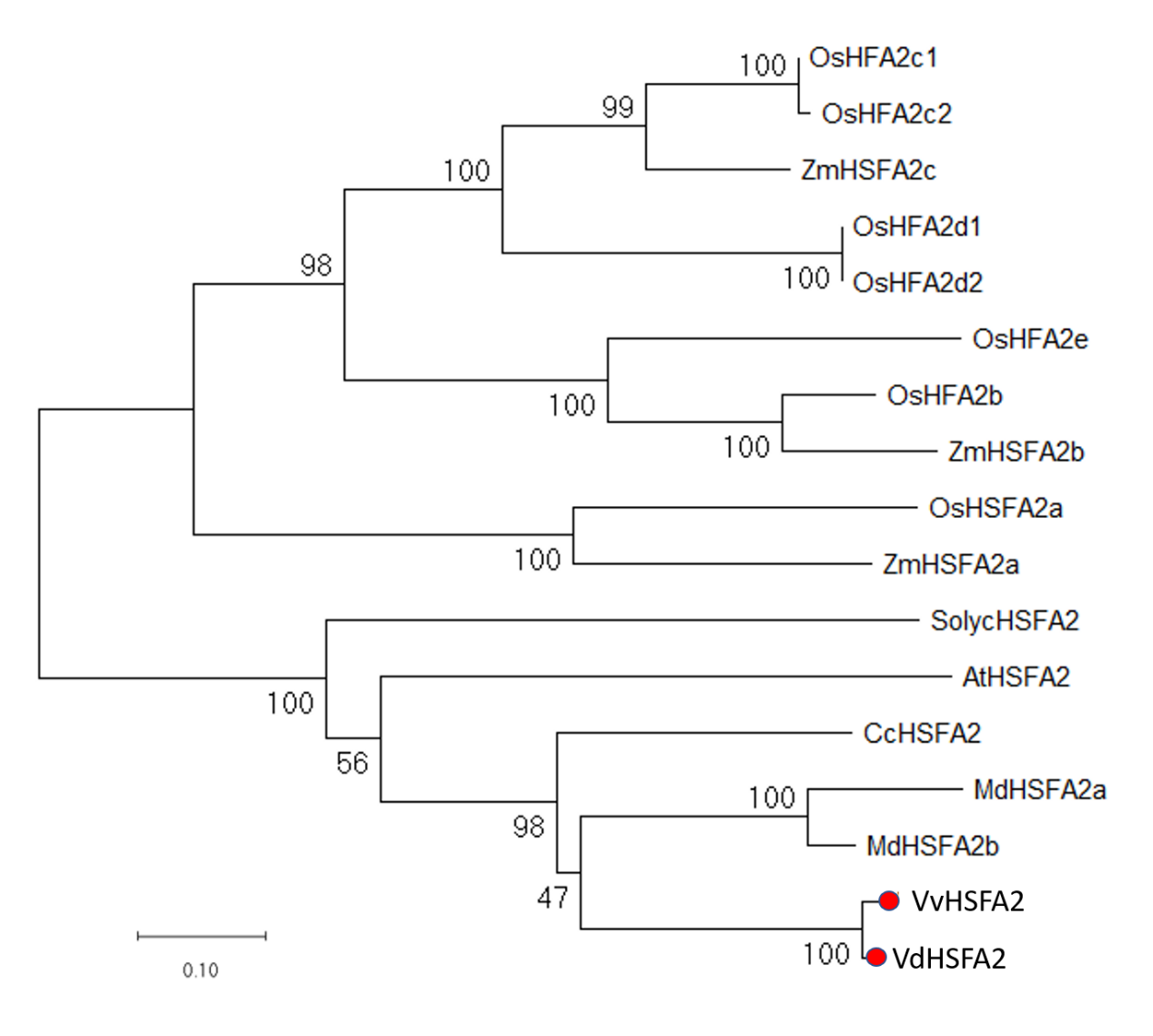
**

**Supplementary Fig.S4 Phylogenetic tree of VvHSFA2 and VdHSFA2 with other plant HSFA2s.**

The tree was conducted using MEGA-X and the statistical reliability of individual nodes of the tree was assessed by bootstrap analysis with 1000 replicates. Os: *Oryza sativa*, Zm: *Zea maize*, solyc: *Solanum lycopersicum*, At: *Arabidopsis thaliana*, Cc: *Citrus clementina*, Md: *Malus domestica*. The sequences were obtained in corresponding species database. VvHSFA2 and VdHSFA2 characterized in this study are shown with two red circles.

**
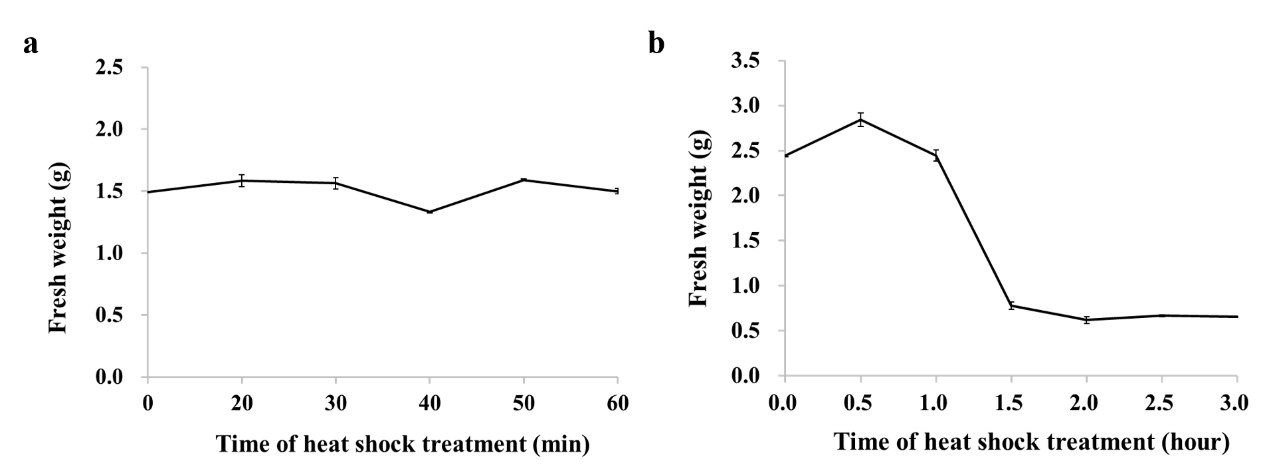
**

**Supplementary Fig. S5 Exploration of heat treatment time and temperature of** **grape suspension cells.**

Fresh weight of grape suspension cells after 45^o^C heat treatment. (a) Fresh weight of grape suspension cells after 45^o^C for different time (minutes). (b) Fresh weight of grape suspension cells after 45^o^C for different time (hours). The fresh weight was measured after heat treatment and recovery 7 d at 25^o^C.Treatment time is shown as X axis. Each point was means (±SE) from three independent biological replicates.

**
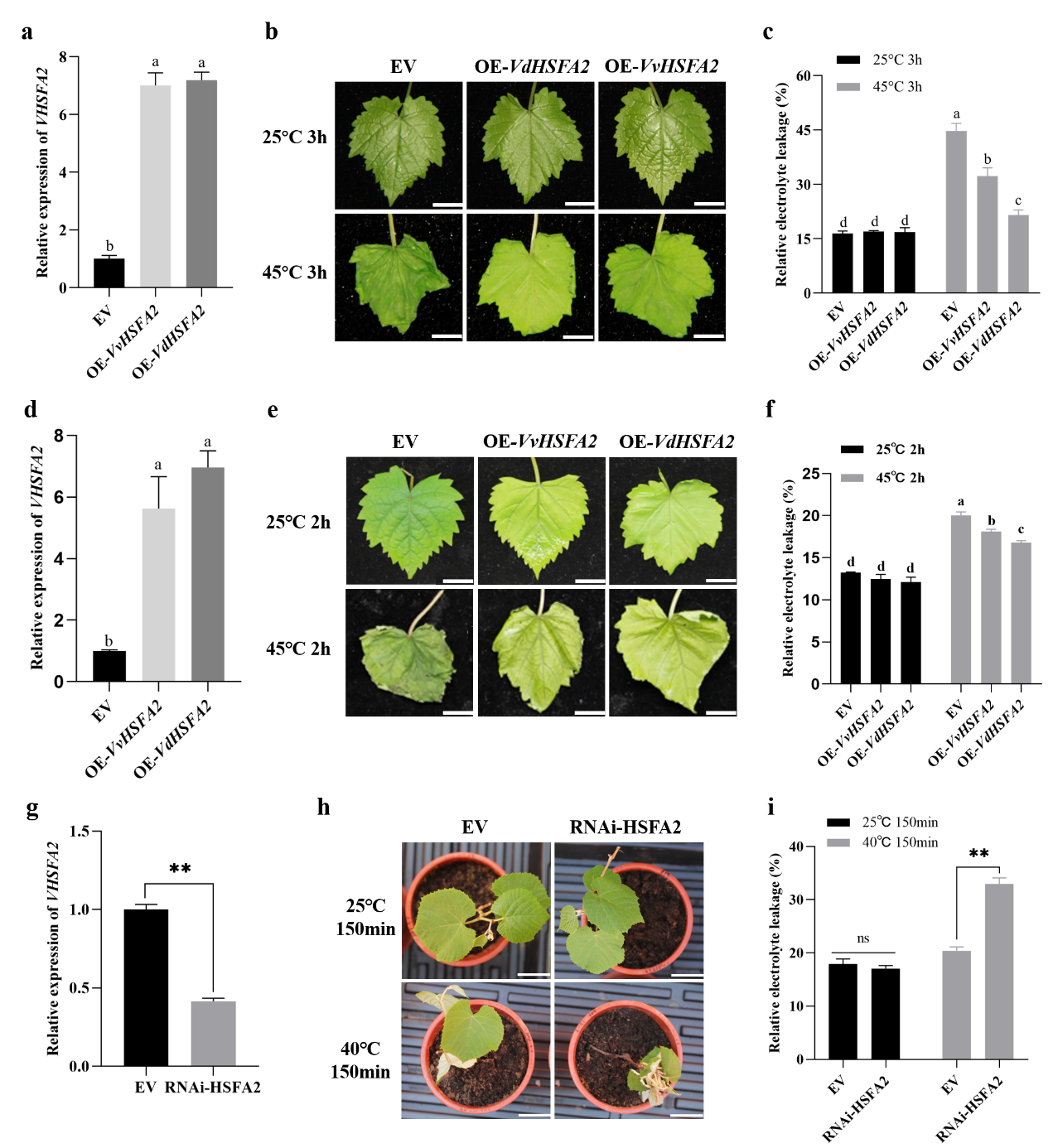
**

**Supplementary Fig. S6 Transiently overexpressed *VvHSFA2* and *VdHSFA2* improved, and interfered *VdHSFA2* weakened thermotolerance of grape plants.**

**a** Expression analysis of *HSFA2* in transiently transgenic ‘Jingxiu’ grape of empty vector (EV), overexpressing *VdHSFA2*(OE-*VdHSFA2*) and *VvHSFA2*(OE-*VvHSFA2*) using RT-PCR. **b** Leaves phenotypes of transiently transgenic ‘Jingxiu’ grape tissue culture plantlets of EV, OE-*VdHSFA2* and OE-*VvHSFA2* before and after 45 ^o^C for 3 h. Bar: 1 cm. **c** Electrolyte leakage rate of EV, OE-*VvHSFA2* and OE-*VdHSFA2* ‘Jingxiu’ plantlets before and after heat treatments. **d** Expression analysis of *HSFA2* in transiently transgenic ‘Summer Black’ grape of empty vector (EV), overexpressing *VdHSFA2*(OE-*VdHSFA2*) and *VvHSFA2*(OE-*VvHSFA2*) using RT-PCR. **e** Leaves phenotypes of transiently transgenic ‘Summer Black’ grape tissue culture plantlets of EV, OE-*VdHSFA2* and OE-*VvHSFA2* before and after 45 ^o^C for 2 h. Bar: 1 cm. **f** Electrolyte leakage rate of EV, OE-*VvHSFA2* and OE-*VdHSFA2* ‘Summer Black’ plantlets before and after heat treatments. **g** Expression analysis of *VdHSFA2* in transgenic *Vitis quinquangularis* plants of empty vector (EV) and RNAi-*VdHSFA2* using RT-PCR. RNAi-*VdHSFA2* was transgenic plants of HSFA2 RNA interference. **h** Phenotypes of EV and RNAi-*VdHSFA2* transgenic plants before and after heat treatments (40^o^C for 150 min). The plantlets grown under 25^o^C was as control. Bar: 3 cm. **i** Electrolyte leakage rate of EV and RNAi-*VdHSFA2* grapevine before and after heat treatments. Data are based on three independent replicates. Duncan test (*P* < 0.05) or Student’s t-test (***P* < 0.01) were used to determine significant differences. ns: not significant.

**
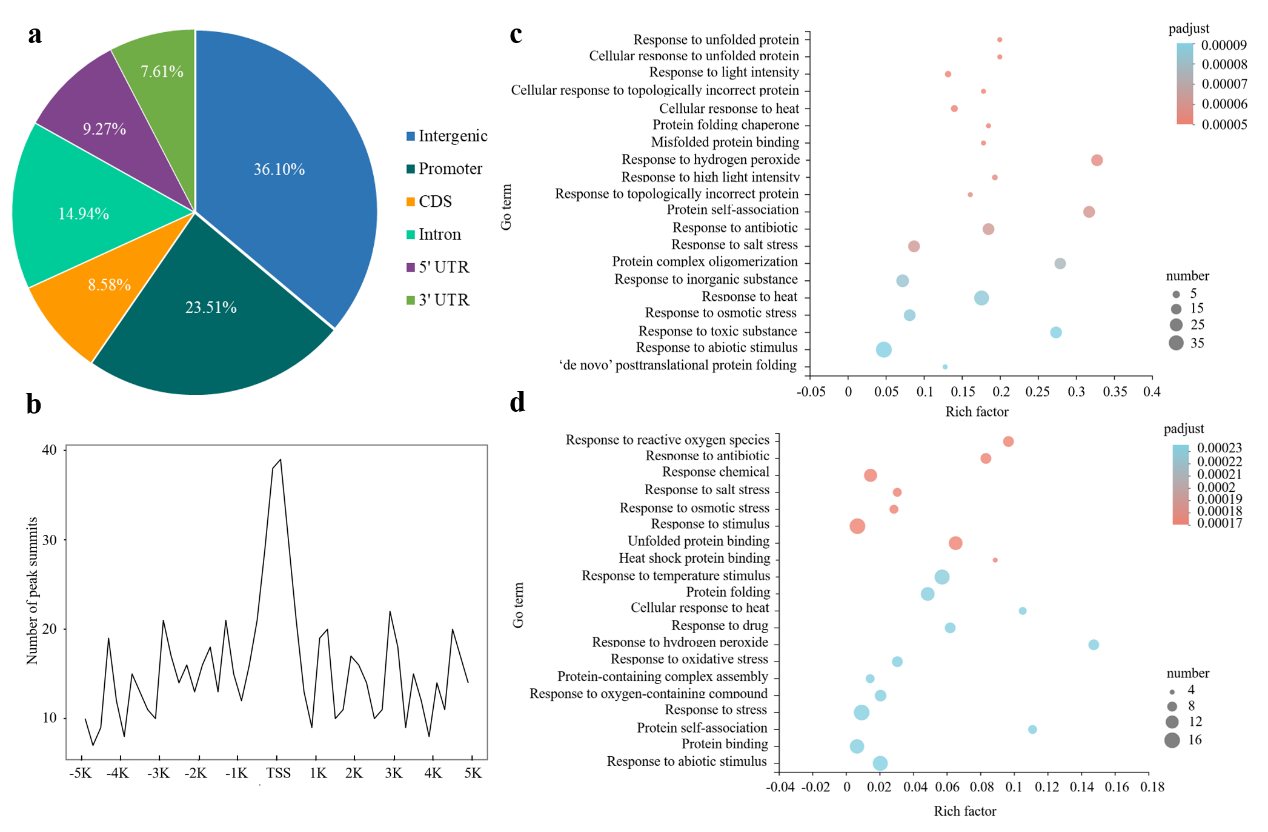
**

**Supplementary Fig. S7 Genome-wide analysis of HSFA2 target genes by ChIP-Seq and RNA-Seq.**

**a** Distribution of VvHSFA2 binding regions in three biological replicates in grape genome. **b** Distribution of VvHSFA2 binding sites around the transcription start sites (TSS) of genes. **c** and **d,** Go classification for genes directly regulated by VdHSFA2 and VvHSFA2, the figure just showing the first 20 enrichments.

**Supplementary Fig. S8 Comparison of *MBF1c* promoter sequences between *Vitis vinifera*** **‘Jingxiu’ and *Vitis davidii* ‘Tangwei’.**

The promoter sequence was cloned 2kb upstream from transcription start site. Alignment was performed using DNAMAN.


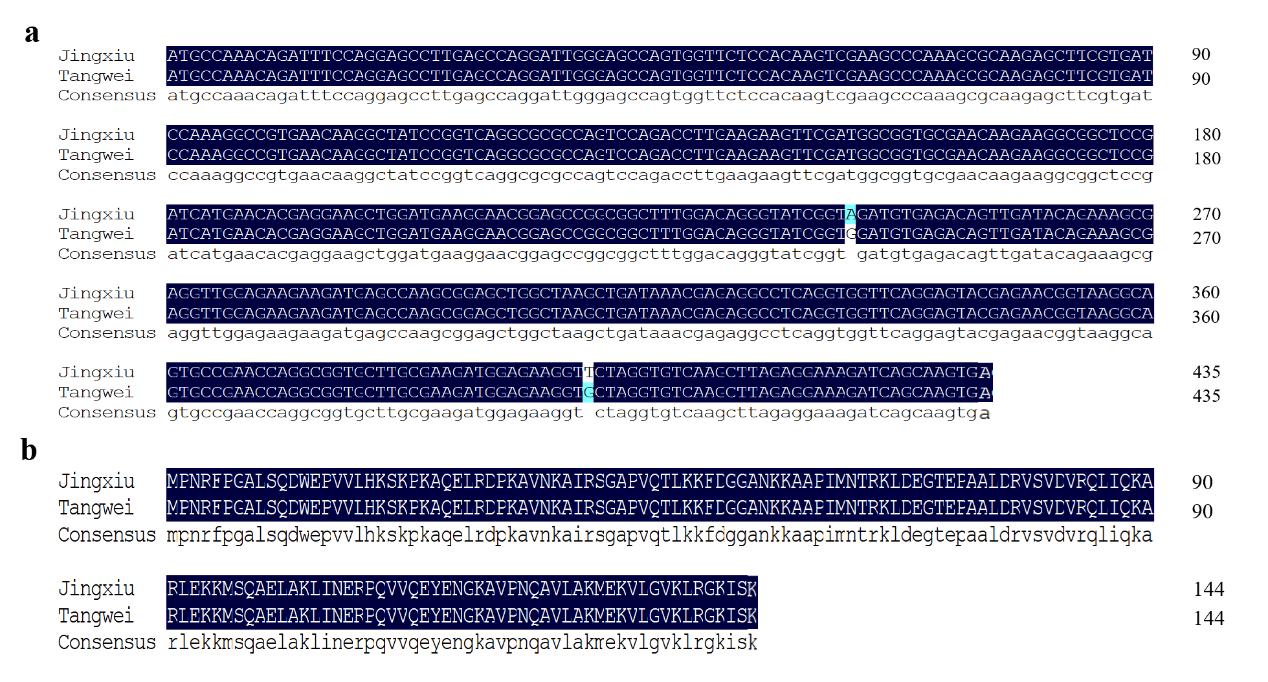


**Supplementary Fig. S9 Comparison of MBF1c coding or amino acids sequences between *Vitis vinifera* ‘Jingxiu’ and *Vitis davidii* ‘Tangwei’.**

**a** Comparison of *MBF1c* coding sequence of ‘Jingxiu’ and ‘Tangwei’. **b** Comparison of MBF1c amino acids of ‘Jingxiu’ and ‘Tangwei’. Alignment was performed using DNAMAN.

**
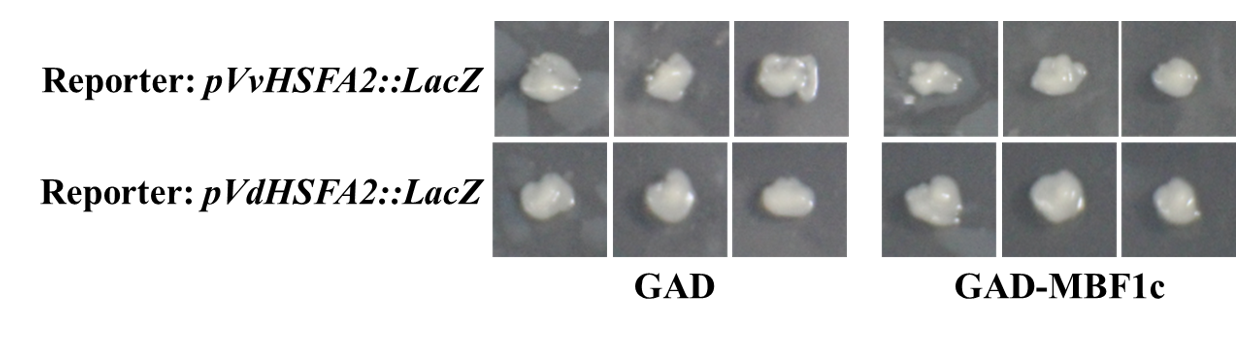
**

**Supplementary Fig. S10 MBF1c can’t bind to *HSFA2* promoters by** **yeast one-hybrid assay.**

Yeast one-hybrid assay of MBF1c binding to *VvHSFA2* and *VdHSFA2* promoter. The coding sequence of MBF1c was fused with GAD vector including Gal4 transcriptional activation domain (AD). Promoter of *VdHSFA2* and *VvHSFA2* was fused with LacZ reporter genes. Constructed vectors were co-transformed into yeast strain EGY48, the transformed yeast were grown on SD/-Trp/–Leu/–Ura/X-α-gal. The transformants with GAD and Reporter: *pMBF1c*::LacZ were used as negative control.

**
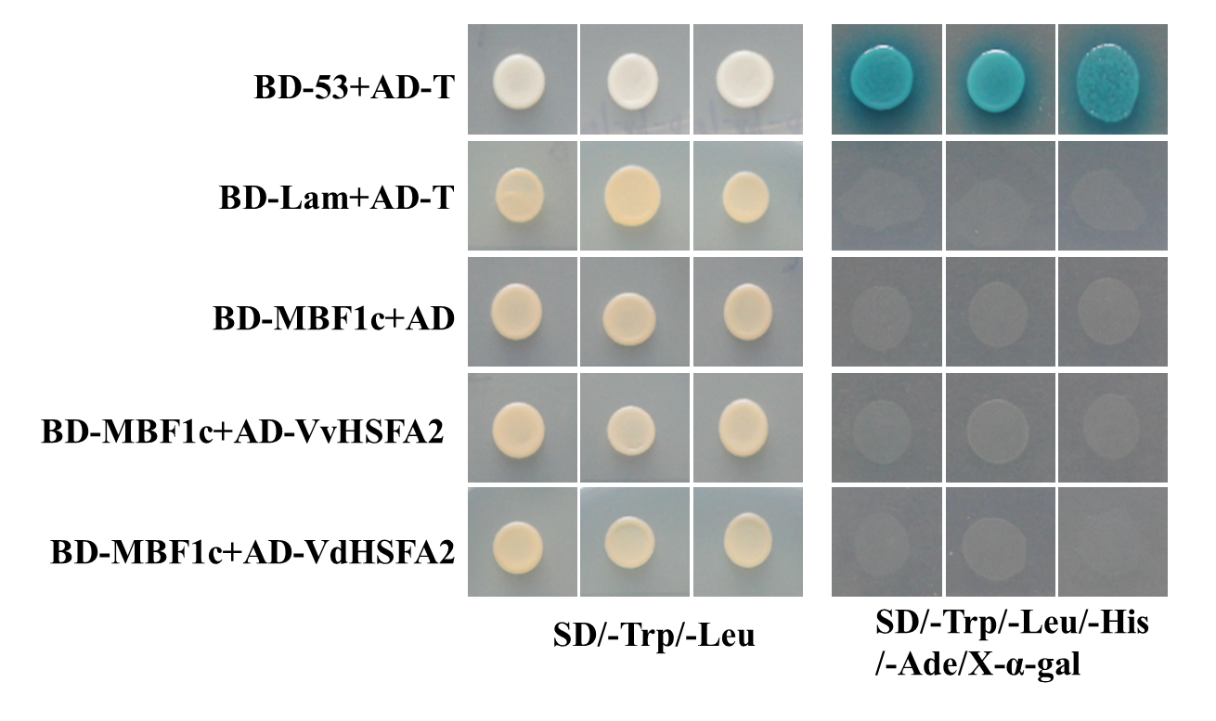
**

**Supplementary Fig. S11 HSFA2 can’t physically interact with MBF1c.**

Yeast two-hybrid assays of the physical interaction of VvHSFA2 and VdHSFA2 with MBF1c. The protein interaction was examined using various combinations of prey and bait vectors. All transformants were spotted on SD/–Trp /– Leu and SD/–Trp /–Leu /–His/–Ade/X-α-Gal selection media. Interactions were determined on the basis of cell growth and cell color.
