# Supplementary figures and images for: Natural variations of HSFA2 enhance thermotolerance in grapevine
Source: Hortic Res. 2022 Nov 10;10(1):uhac250. doi: 10.1093/hr/uhac250 (PMC9832954; doi:10.1093/hr/uhac250)

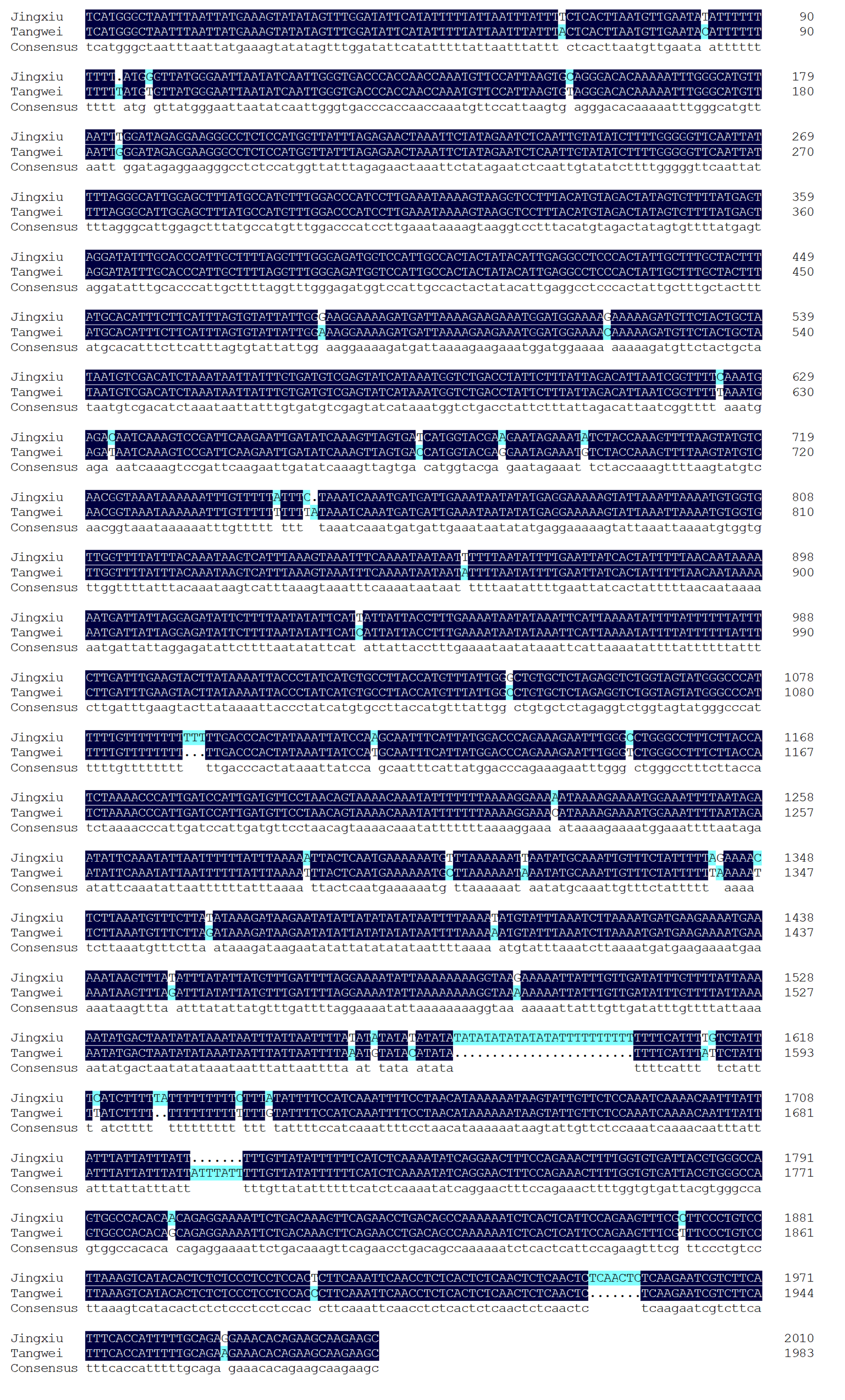

Supplement: Web_Material_uhac250 [file web_material_uhac250.zip › Supplementary Fig. 1.tif]

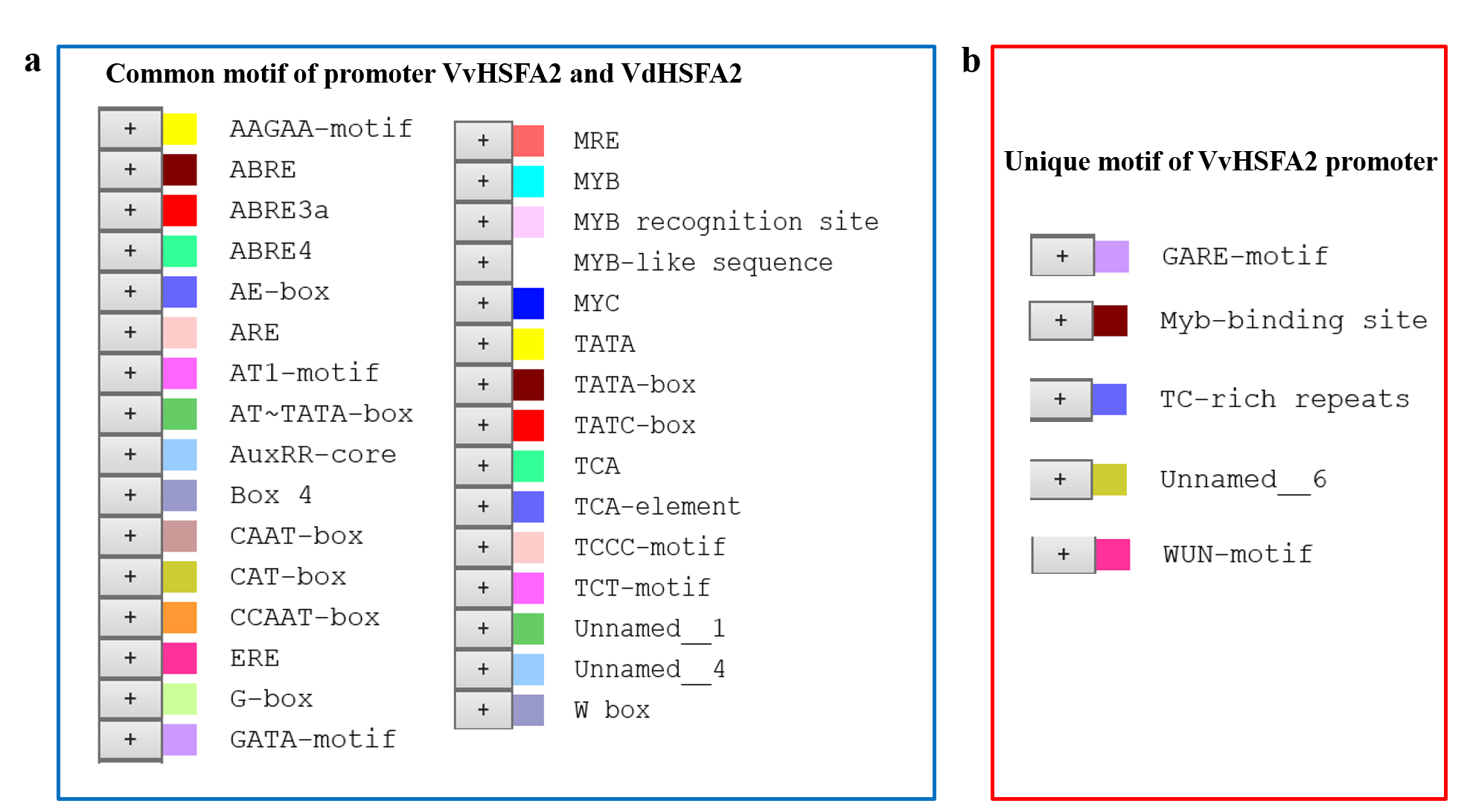

Supplement: Web_Material_uhac250 [file web_material_uhac250.zip › Supplementary Fig. 2.tif]

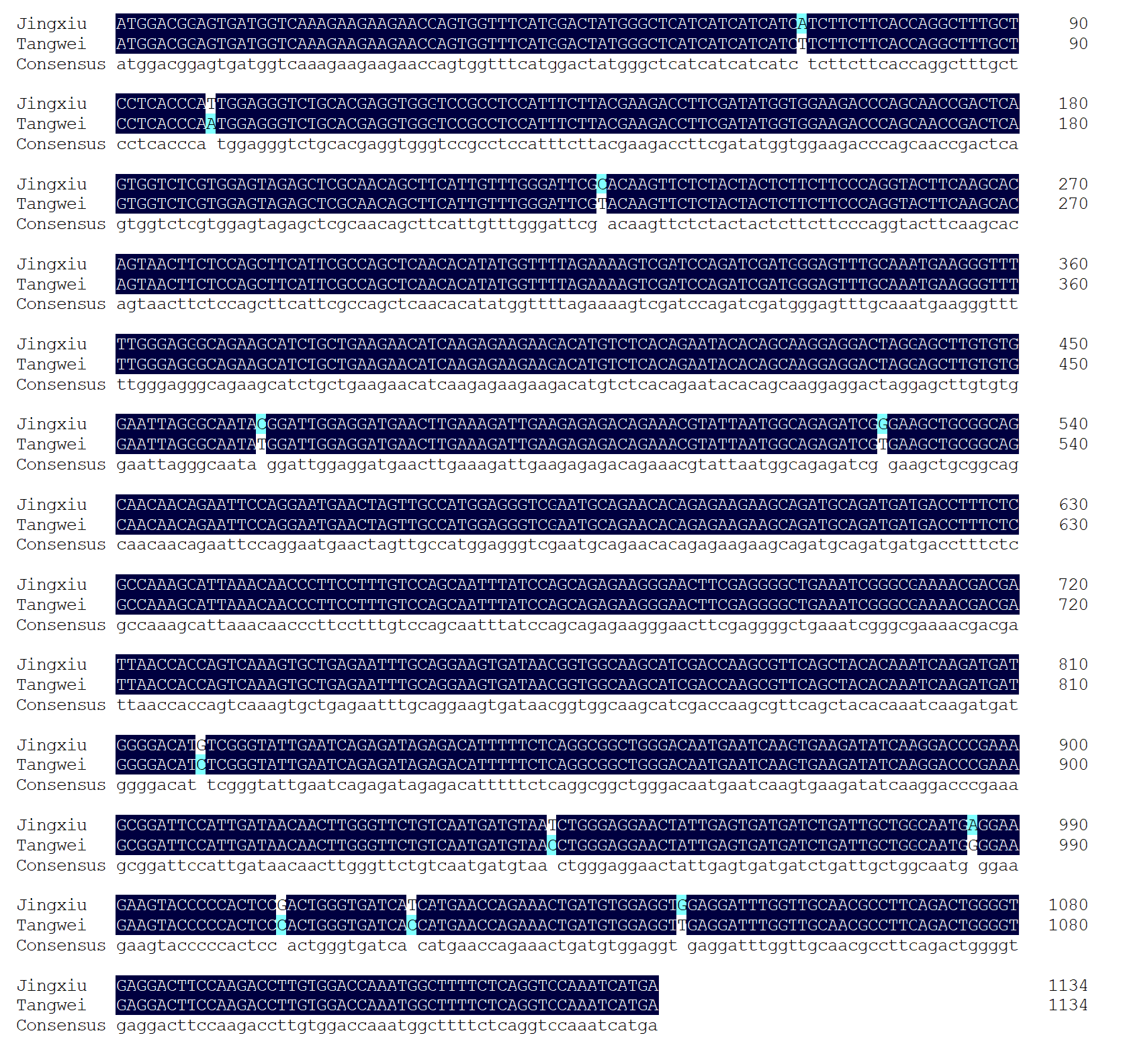

Supplement: Web_Material_uhac250 [file web_material_uhac250.zip › Supplementary Fig. 3.tif]

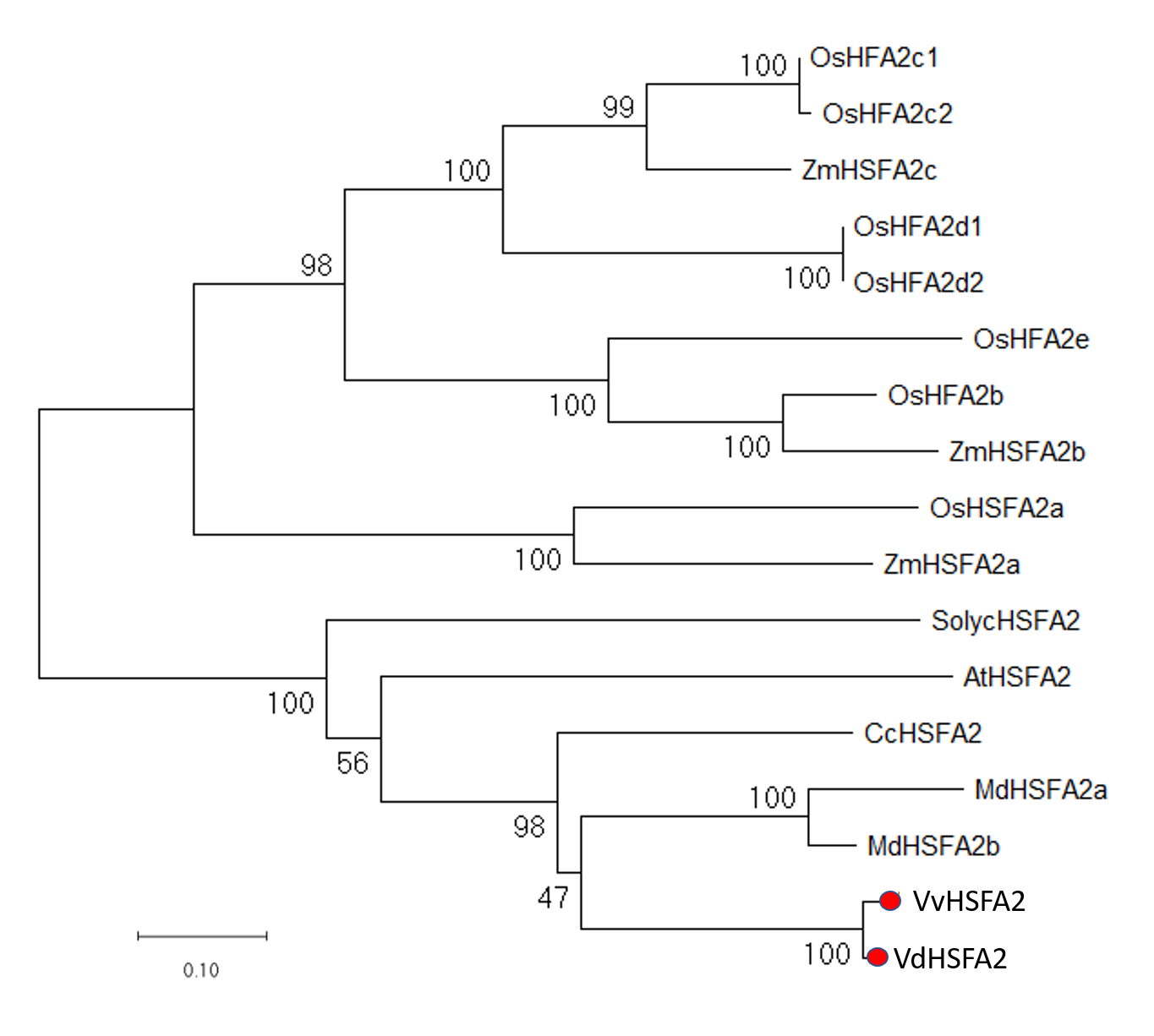

Supplement: Web_Material_uhac250 [file web_material_uhac250.zip › Supplementary Fig. 4.tif]

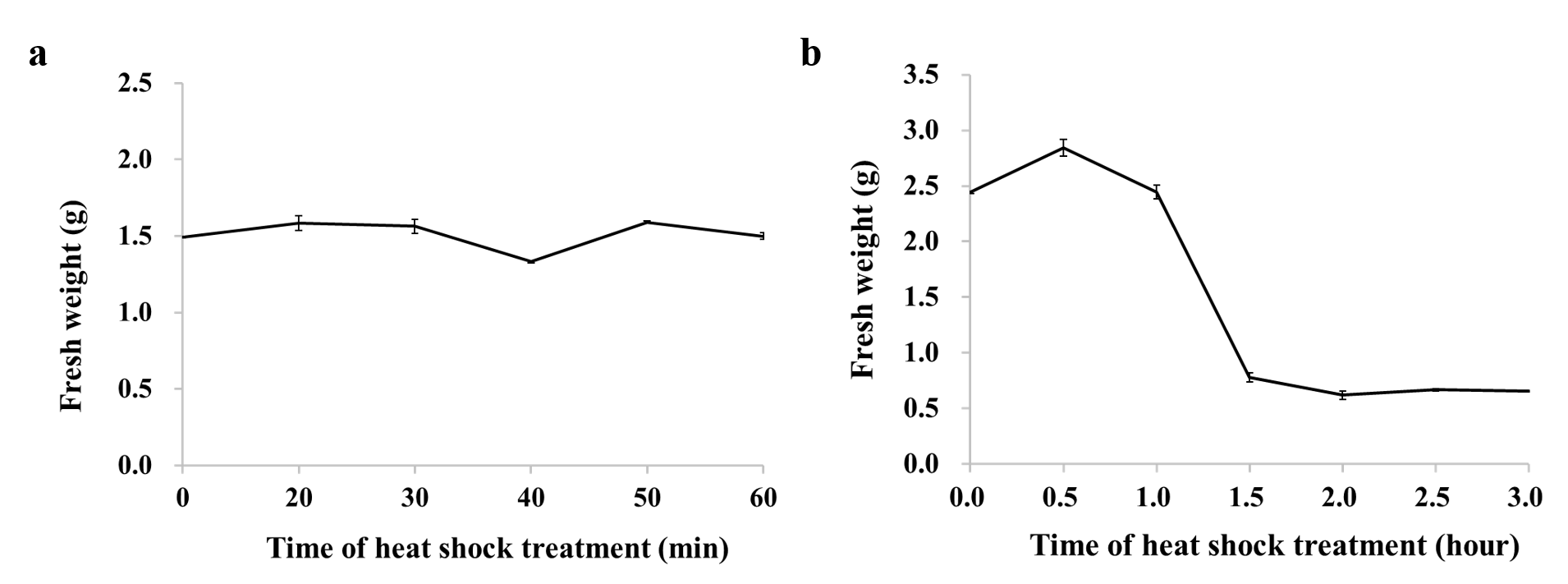

Supplement: Web_Material_uhac250 [file web_material_uhac250.zip › Supplementary Fig. 5.tif]

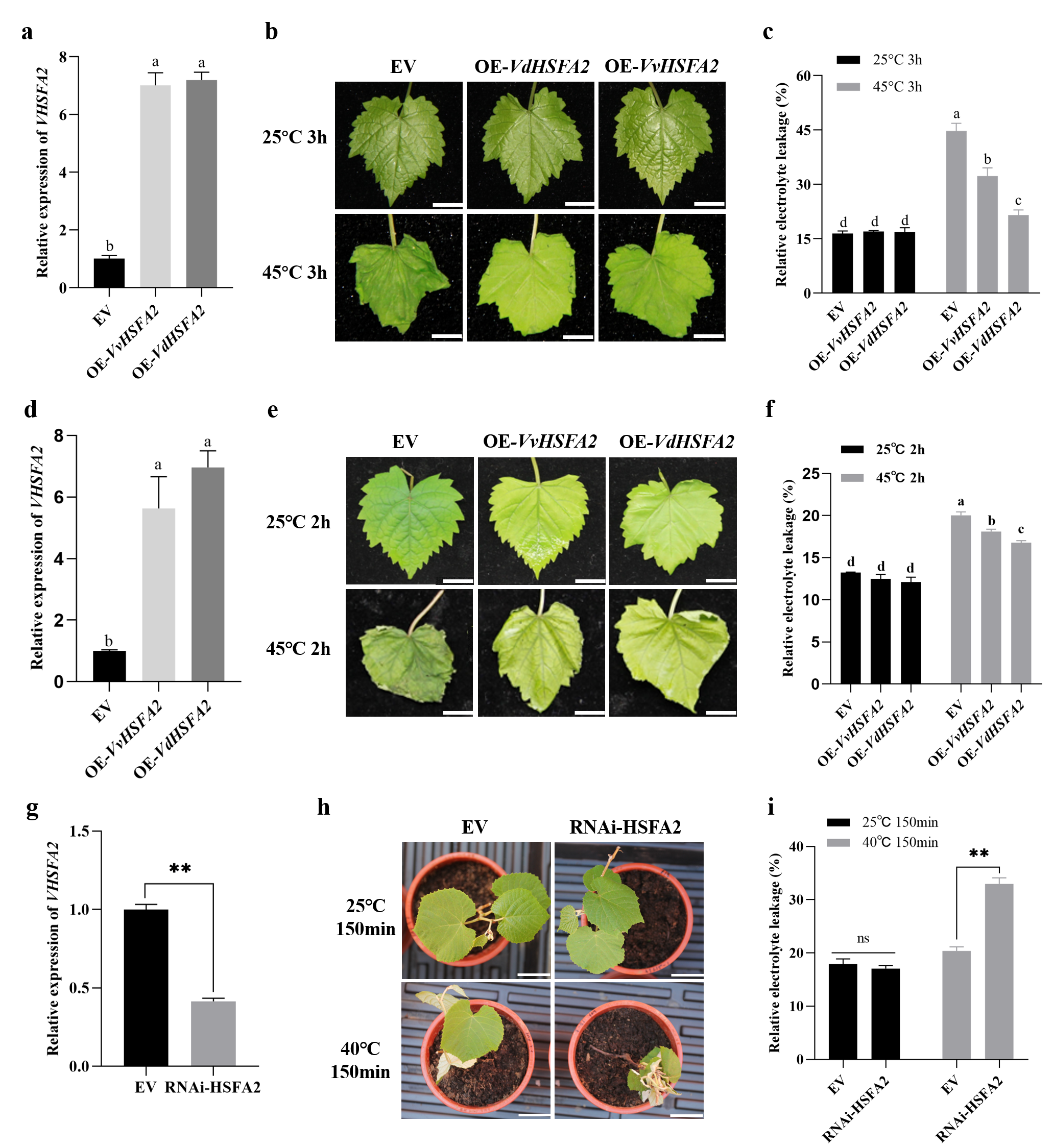

Supplement: Web_Material_uhac250 [file web_material_uhac250.zip › Supplementary Fig. 6.tif]

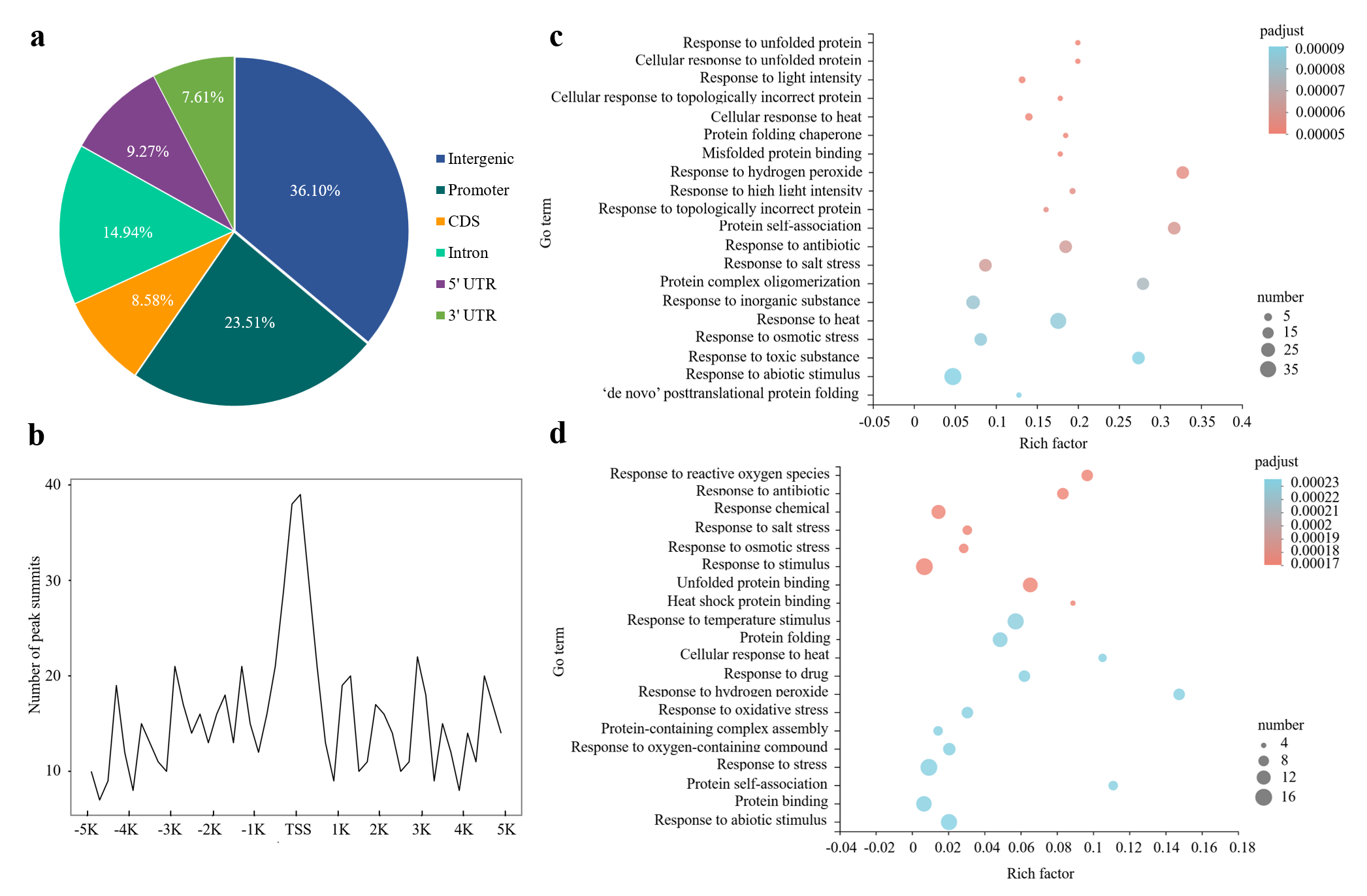

Supplement: Web_Material_uhac250 [file web_material_uhac250.zip › Supplementary Fig. 7.tif]

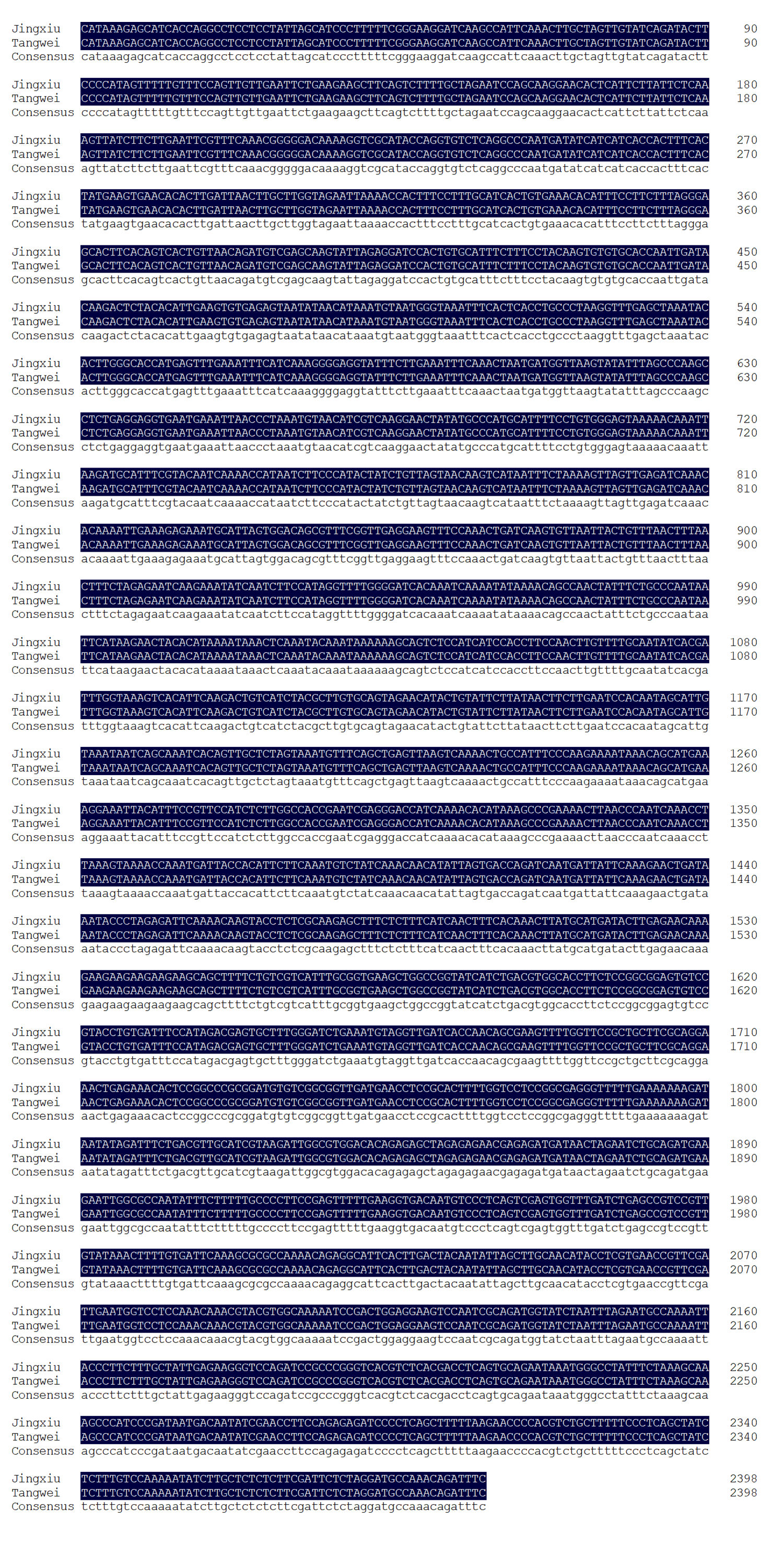

Supplement: Web_Material_uhac250 [file web_material_uhac250.zip › Supplementary Fig. 8.tif]

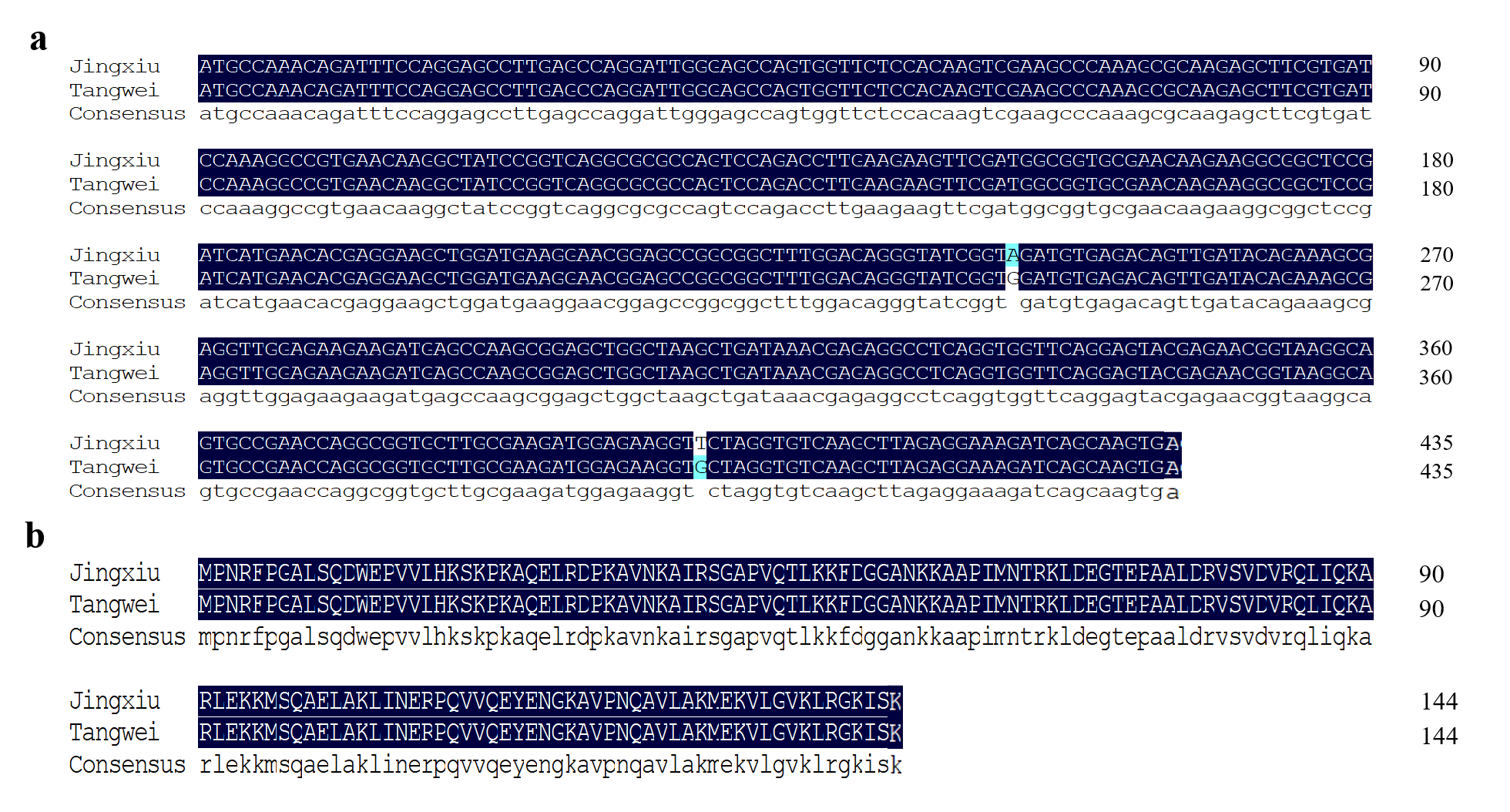

Supplement: Web_Material_uhac250 [file web_material_uhac250.zip › Supplementary Fig. 9.tif]

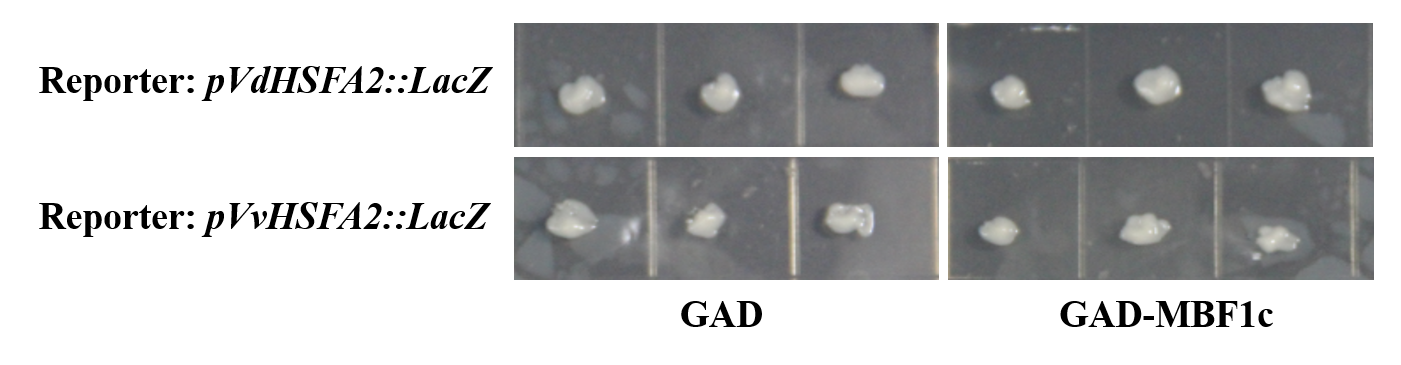

Supplement: Web_Material_uhac250 [file web_material_uhac250.zip › Supplementary Fig. 10.tif]

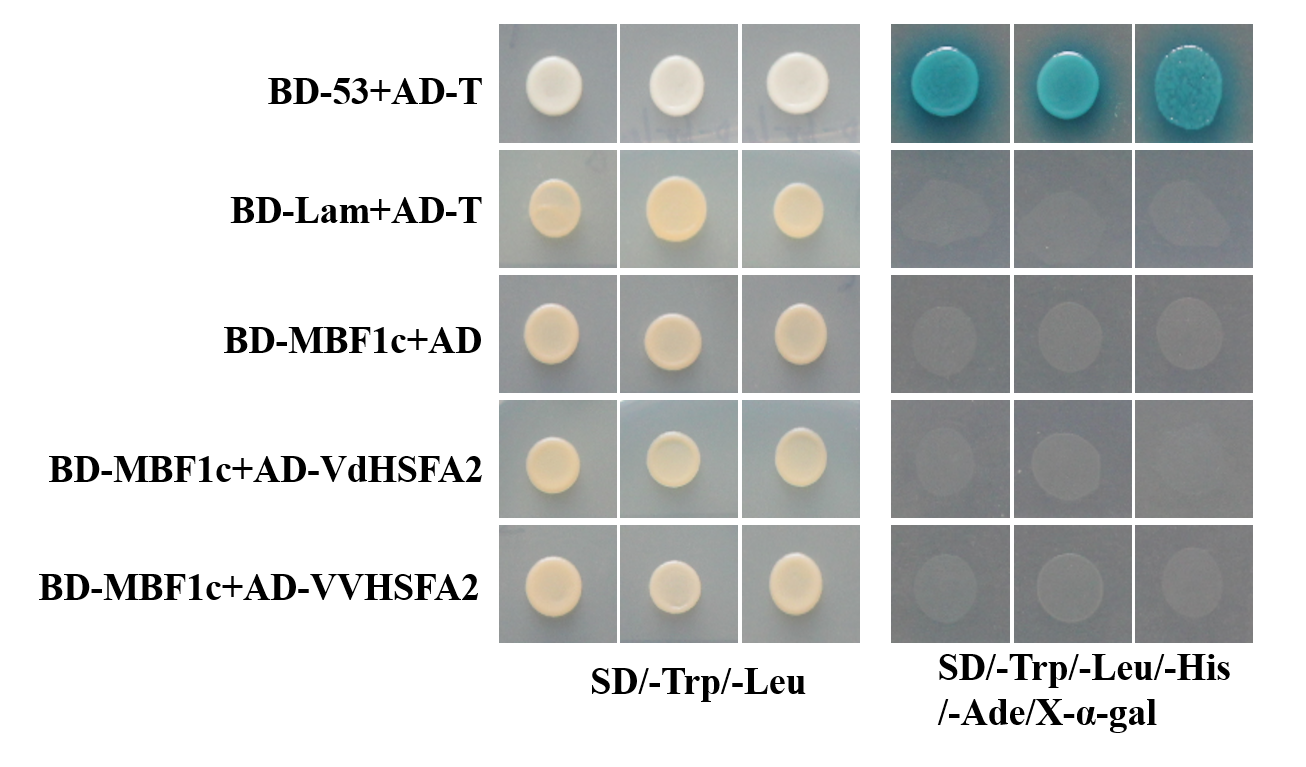

Supplement: Web_Material_uhac250 [file web_material_uhac250.zip › Supplementary Fig. 11.tif]
